# CD81 fusion alters SARS-CoV-2 Spike trafficking

**DOI:** 10.1128/mbio.01922-24

**Published:** 2024-08-14

**Authors:** Allaura S. Cone, Yijun Zhou, Ryan P. McNamara, Anthony. B. Eason, Gabriel F. Arias, Justin T. Landis, Kyle W. Shifflett, Meredith G. Chambers, Runjie Yuan, Smaranda Willcox, Jack D. Griffith, Dirk P. Dittmer

**Affiliations:** 1 Department of Microbiology and Immunology, The University of North Carolina at Chapel Hill, Chapel Hill, North Carolina, USA; 2 Lineberger Comprehensive Cancer Center, The University of North Carolina at Chapel Hill, Chapel Hill, North Carolina, USA; 3 Ragon Institute of MGH, MIT and Harvard, Cambridge, Massachusetts, USA; 4 Department of Biochemistry and Biophysics, The University of North Carolina at Chapel Hill, Chapel Hill, North Carolina, USA; Icahn School of Medicine at Mount Sinai, New York, New York, USA

**Keywords:** exosome, SARS-CoV-2, coronavirus, Spike, CD81, CD63, TSPAN, extracellular vesicles, tetraspanins, dSTORM

## Abstract

The severe acute respiratory syndrome coronavirus 2 (SARS-CoV-2) pandemic caused the biggest public health crises in recent history. Many expect future coronavirus introductions into the human population. Hence, it is essential to understand the basic biology of these viruses. In natural infection, the SARS-CoV-2 Spike (S) glycoprotein is co-expressed with all other viral proteins, which modify cellular compartments to maximize virion assembly. By comparison, most of S is degraded when the protein is expressed in isolation, as in current molecular vaccines. To probe the maturation pathway of S, we redirected its maturation by fusing S to the tetraspanin protein CD81. CD81 is a defining constituent of extracellular vesicles (EVs) or exosomes. EVs are generated in large numbers by all cells, extruded into blood and lymph, and transfer cargo between cells and systemically (estimated 10^12^ EVs per mL plasma). EVs, like platelets, can be transfused between unrelated donors. When fusing the proline-stabilized form of strain Delta S into the flexible, large extracellular loop of CD81 rather than being degraded in the lysosome, S was extruded into EVs. CD81-S fusion containing EVs were produced in large numbers and could be isolated to high purity. Purified CD81::S EVs bound ACE2, and S displayed on individual EV was observed by cryogenic electron microscopy (EM). The CD81::S-fusion EVs were non-toxic and elicited an anti-S trimer and anti-RBD antibody response in mice. This report shows a design path to maximize viral glycoprotein assembly and release without relying on the co-expression of potentially pathogenic nonstructural viral proteins.

**IMPORTANCE:**

The severe acute respiratory syndrome coronavirus 2 pandemic caused the biggest public health crises in recent history. To understand the maturation pathway of S, we fused S to the tetraspanin protein CD81. The resulting molecule is secreted in extracellular vesicles and induces antibodies in mice. This may be a general design path for viral glycoprotein vaccines.

## INTRODUCTION

The rapid development and deployment of severe acute respiratory syndrome coronavirus 2 (SARS-CoV-2) vaccines represent this decade’s largest public health success. Current coronavirus disease 2019 (COVID-19) vaccine platforms have demonstrated the efficacy of recombinant single antigen vaccination, whether driven by RNA lipid nanoparticles, protein nanoparticles, or recombinant vector platforms (1–6). The COVID-19 experience reminded the world of the need to advance our understanding of virus biogenesis, pathogenesis, and immunity. These research efforts are collectively characterized under “pandemic preparedness” (7, 8). Fortunately, the coronavirus Spike (S) glycoproteins are inherently immunogenic. Unfortunately, immunity in the form of neutralizing antibody titers to S is short-lived and strain-specific, counteracted by a rapidly evolving pool of human SARS-CoV-2 lineages (9). Current coronavirus vaccines prevent disease, not infection; they do not induce sterilizing immunity to protect from future outbreaks.

This report adds to the basic understanding of coronavirus virion biogenesis by dissecting and redirecting the S protein maturation pathway. Engineering a viral-host protein fusion molecule redirected the natural intra-cellular biogenesis and maturation pathway of SARS-CoV-2 S. This study lies at the intersection of two fields: virology and extracellular vesicle (EV) research. Hence, it is useful to review both.

SARS-CoV-2 entered the human population in late 2019 (10–13). SARS-CoV-2 is a member of the positive-sense single-stranded RNA-based genus *Sarbecovirus* within the family *Coronaviridae* (14). The virus is enveloped, and like other coronaviruses, SARS-CoV-2 uses the envelope Spike (S) protein for cell attachment and entry. The S protein contains a receptor binding domain (RBD), amino acids Arg319–Phe541, which binds to the peptidase domain of the human angiotensin-converting enzyme (ACE2) (15, 16). Soluble ACE2 or ACE2 mimics block SARS-CoV-2 entry by binding to the RBD domain of the S protein (17, 18) and antibodies directed against the RBD domain block virus entry.

S exists as a homo-trimer. Each monomer unit (S0) is glycosylated, first in the ER and then in the Golgi. It has a monomeric molecular mass of >180 kDa (19, 20). The intact virus particle contains between 20 and 40 homotrimers on the surface, resulting in a crownlike structure when viewed by electron microscopy (EM) (21, 22). S exists in a prefusion and a post-fusion form. The trimer adopts intermediate structures as well (15, 23). Conformational changes are necessary to rotate the three RBD domains on the trimer upward before they become accessible to ACE2. An all-RBD-down conformation is inaccessible to ACE2 and is referred to as “closed” (24). In this study, the binding to ACE2 is considered evidence for correct trimer folding.

The inactive precursor S0 is cleaved by cellular furin proteases at the polybasic site 679–684 PRRAR.S into two subunits, S1 and S2, before trafficking to the cell surface. While this exact sequence is not shared, other coronaviruses have similar furin cleavage sites (25–27). The S1 subunit contains the RBD. S1 and S2 trimerize to yield a ∼600 kDa complex of six non-covalently linked subunits, with the S1 subunits forming a “cap” atop the S2 stem. Therefore, S1 and S2 subunits are present in equal proportions on the virion particle. The S subunits have different apparent molecular weights on denaturing SDS polyacrylamide gels depending on processing and assembly events. S1 isolated from the cell membrane of virus-negative, transfected HEK293 cells migrates at 110 kDa; the furin-cleaved S2 unit at 90 kDa. S2 undergoes an additional cleavage event by the TMPRSS2 protease at the S2' (811- KSPKR.S) site (28) at the target cell surface. Either TMPRSS2 and/or endosomal–lysosomal proteases expose the fusion peptide and facilitate virion to host membrane fusion and release of the capsid into the cytoplasm.

SARS-CoV-2 S maturation through cellular compartments is well studied (reviewed in reference 29). Wild-type SARS-CoV-2 S undergoes glycosylation in the Golgi and is sorted into the lysosome; furin cleavage occurs in the trans-Golgi network (TGN). This pathway is conserved across the *betacoronaviridae* family, although the molecular sorting motifs are not (20). SARS-CoV-2 S does not have a linear lysosomal sorting signal. During natural infection, the virion consisting of Spike (S), membrane (M), nucleocapsid (N), envelope (E), and the viral genomic RNA is assembled in the ER. The virions and free S protein do not move independently into and beyond the TGN, as is the case for other viruses. In SARS-CoV-2, the nonstructural (NS) orf3A protein facilitates the anterograde movement of the lysosomes to the plasma membrane (30). How recombinant S, e.g., as expressed from current mRNA vaccine platforms, is trafficked in the absence of the coronavirus ancillary proteins is unknown (31).

The first mutation to emerge during the SARS-CoV-2 pandemic was the D614G substitution. D614G viruses rapidly replaced the ancestral wild-type strain in a, at the time, virus-naïve population (32, 33). D614G-containing S proteins result in a higher proportion of S trimers with the RBD subunit in the “up” position, i.e., ready to bind ACE2 (34–36). In addition, D614G increases S trafficking into the lysosomal pathways (19). D614G is a natural allosteric mutation, one could say the most dramatic, as it was selected first, and it is retained in all subsequent strains, including recombinant XBB lineages (37, 38).

The first engineered mutations in S were proline substitution mutants in the S2 “stalk.” These increase the stability of the prefusion form for several Coronaviruses, including SARS-CoV-2 (23, 24, 39). Stabilized forms of SARS-CoV-2 S may contain two, four, or six prolines and additional substitution mutations. The proline mutations are located in the turns connecting the central helices in S2, which becomes a single, elongated helix in the post-fusion conformation. They inhibit the conformational change to the post-fusion form. The secreted, soluble “HexaPro” variant in the wild-type background produced a mixture of complexes: one with a single RBD in the up conformation and the other with two RBDs in the up conformation (24). To date, no one has evaluated the cellular trafficking of these stabilized S proteins to the cell membrane, into virions, or EVs.

EVs are small membrane-bound particles critical for cell-to-cell communication (reviewed in references 40, 41). EVs package various cargo, such as proteins, lipids, metabolites, and nucleic acids. EVs are secreted by all cell types and found in all bodily fluids evaluated to date. They are utilized extensively by human viruses (reviewed in reference 42). Some have called them the “Trojan horses” for viruses (43). Exosomes are an EV subgroup, ranging in size from 40 to 150 nm. Some EVs originate by the inward budding of endosomes into the multivesicular body (MVB) (41, 44), and others originate at the cell surface akin to retrovirus budding (45, 46). Irrespective of origin, EV surfaces are marked with one or more tetraspanin (TSPAN) proteins on their surface, most prominently CD9, CD63, and CD81.

TSPAN are four-pass transmembrane proteins (47). They interact with lipids, such as ceramide or cholesterol (48), and accessory proteins, such as ALIX and Syntenin-1 (49). TSPAN contains a small extracellular loop (SEL) and a large extracellular loop (LEL) (50). The LEL of tetraspanins has a conserved and variable domain (51). The variable domain is thought to determine the protein’s interaction partners and signaling capacity (52). The LEL, but not the SEL, is flexible and can undergo conformational changes to accommodate the aggregation of multiple tetraspanins in quaternary complexes (homo or heteromeric) on the cell surface and EV membrane.

CD81, also known as TAPA-1 (target of anti-proliferative antibody 1), is a TSPAN (53, 54). CD81 is palmitoylated and has a short, intracellular C-terminus: -KRNSSVY-COOH (55). On CD81, the LEL sits atop the SEL, restricting access. On CD81, the allosteric movements of the LEL are very pronounced and orchestrated upon cholesterol binding between the four helices of the transmembrane region (48). The variable region δ-domain of CD81 LEL is vital for forming tetraspanin webs on the cell surface (56, 57). On B cells, CD81 forms a complex with CD19, CD21/CR2, and CD225. In fact, CD81 is a trafficking factor that chaperones CD19 to the plasma membrane. In the liver, CD81 binds to the hepatitis C virus (HCV) E2 envelope protein and is required for efficient HCV entry (58, 59).

We hypothesized that we could direct D614G SARS-CoV-2 Spike (^D614G^S_WA1_) trafficking from the lysosomal pathway (60), where a lot of the protein is degraded unless NS orf3A is co-expressed, into EVs. To test this hypothesis, we fused S to CD81. This changed the biosynthesis trafficking of S to become cell surface and EV associated. S decorated the surface of the EVs as trimers, as ascertained by cryogenic EM (cryo-EM) and super-resolution microscopy. The S-EVs bound ACE2 under physiological conditions, evidencing that one or more RBD domains were in the ACE2 accessible “up” position. S-EVs elicited an anti-S antibody response in mice.

## MATERIALS AND METHODS

### Constructs

**Fig 1:**
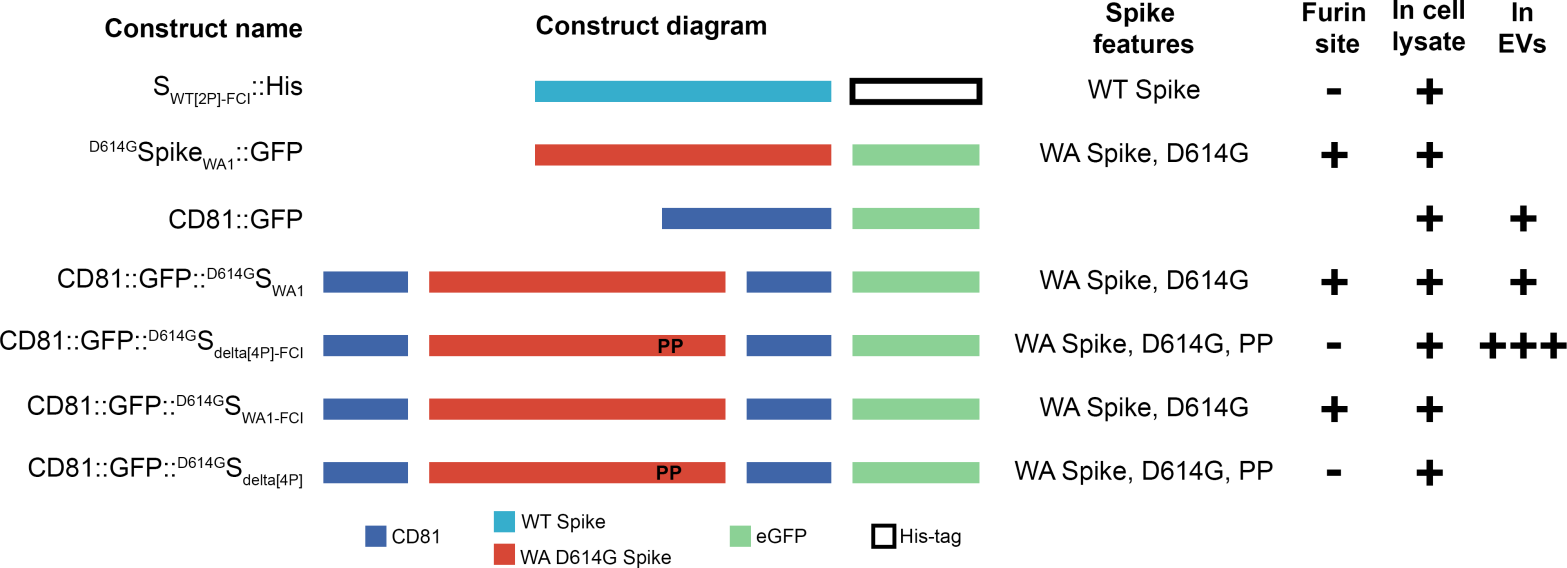
Map of constructs that lists the names, a diagram, the features, and if the S protein has an intact furin cleavage insert. Additionally, we also list if the construct was tested in cell lysate and if we have seen the construct present on EVs using dSTORM. Blue is for CD81, red is for ^D614G^S, blue is for wild-type S, green is for GFP, and open box is for the His tag.

The CD81-Spike recombinant plasmids were designed by extracting sequences from the indicated database and built into the pcDNA3.1(+) plasmid: CD81 (NCBI, CCDS, 7734.1), green fluorescent protein (GFP) (Addgene Plasmid #62964), S (GenBank, MT565498.1), Spike Delta variant (GISAID, Accession ID #EPI_ISL_2710011, add G142D, R158G, and D950N). The stabilized S delta variant was built by introducing six proline stabilizing mutations (K986P, V987P, F817P, A892P, A899P, and A942P) (24) into the S delta variant sequence described previously. Plasmids were synthesized by Genscript and confirmed by restriction enzyme digestion and Sanger sequencing. Complete sequences are available in GenBank. The constructs used in this study are ^D614G^Spike_WA1_::GFP (pDD3511), CD81::GFP (pDD3513), CD81::GFP::^D614G^S_WA1_ (pDD3515), CD81::GFP::^D614G^S_delta[4P]-FCI_ (pDD3521), CD81::GFP::^D614G^S_WA1-FCI_ (pDD3800), and CD81::GFP::^D614G^S_delta[4P]_ (pDD3801). Here, “::” refers to a protein-protein fusion. The secreted form of stabilized S protein expression plasmid S_WT[2P]-FCI_::His (pDD3503) was a gift from Dr. Florian Krammer (61). SARS-CoV-2 orf3a::mCherry plasmid was ordered from Addgene (plasmid number 165138). Constructs are summarized in Table S1 and Fig. 1.

### Cell culture

Human osteosarcoma (U-2 OS) and HEK293T cell lines were obtained from ATCC (Cat# HTB-96 and CRL-3216) and were certified as mycoplasma free. Cells were grown in Dulbecco’s modified Eagle medium (DMEM; Thermo Fisher, Cat# 21013024) supplemented with 10% exosome-free fetal bovine serum (VWR, Cat# 97068-085), 20 mM of L-glutamine (Gibco 25030-081), 100 units/mL penicillin, and 100 µg/mL streptomycin solution (Gibco, Cat# 15140-122). Cells were maintained at 37°C and 5% CO_2_ in a HERAcell 150i incubator (Thermo Fisher, Cat# 50116050). Cells were transfected with the construct of choice using Lipofectamine-2000 or Lipofectamine-3000 (Thermo Fisher, Cat# 11668019 and L3000015) diluted in OptiMEM (Gibco, Cat# 31985062). After 24 hours, the cells were placed in the selection medium (G418 at 250 µg/mL). After selection, single-cell sorting using the FACS Aria II BSL2 was performed by the University of North Carolina at Chapel Hill (UNC) Flow Cytometry Core. Two independent clones were synthesized and examined for each design. These clones were expanded in culture to create a homogeneous population with decreased heterogeneity in the EVs produced.

### Purification of EVs

Cells were seeded in 20 mL medium and grown to 80% confluency before the addition of another 80 mL medium to start conditioning for EV harvest. Cell viability was monitored by observing morphology and medium PH. After 72 hours, conditioned medium was collected and centrifuged at 1,000 × g for 5 minutes. Next, medium was filtered through a 0.45 µm (Genesee 25-230) and a 0.22 μm filter (Genesee 25-229). The medium was then concentrated by tangential flow filtration. An ÄKTA Flux Tangential Flow Filtration System with a MidGee Hoop hollow fiber (Cytiva, 750 kDa MWCO, UFP-750-C-H24LA) was equilibrated in sterile 1× phosphate-buffered saline (PBS; Gibco, Cat: 14190144). Ultrafiltration to a final volume of 1/10 starting volume was completed using a feed flow of 34 mL/min at a transmembrane pressure of 27 psi. Concentrated media were then incubated with a 4% final concentration of polyethylene glycol (PEG; Fisher BioReagents BP233-1) overnight at 4°C. The next day, the medium was centrifuged at 1,000× g for 1 hour at 4°C. The supernatant was removed, and the crude PEG pellet was resuspended in cold PBS with RNase (Thermo Fisher, Cat# EN0531), DNase (Promega, Cat#: M6101), and/or Cell Mask Red dye (Thermo Fisher C10046) and incubated overnight at 4°C. Finally, the resuspension was purified through a HiTrap CaptoCore700 column (Cytiva 17548151). The EVs were kept at −80°C until use.

### Nanoparticle tracking analysis of EV

Nanoparticle tracking analysis (NTA) was done as previously described (62). Briefly, ZetaView Nanoparticle Tracking Analyzer (Particle Metrix, PMX‐120) was used to determine the size and concentration of particles. Before analysis, the instrument was standardized using 100 nm Nanosphere beads (Thermo Scientific 3100A) with a sensitivity of 65 and a shutter of 100 (ZetaView 8.04.02). Samples were diluted in ddH_2_O, with 50 to 200 particles per window. Data were acquired with sensitivity at 88, shutter at 70, maximum area at 220, minimum area at 5, and minimum brightness at 20, with each measurement run for three cycles, with three measurements.

### Western blot analysis

Cell lysates were lysed in a radioimmunoprecipitation assay (RIPA) buffer, and EVs were lysed in a strong lysis buffer (5% SDS, 10 mM EDTA, 120  mM Tris-HCl [pH 6.8], and 8 M urea). Protein concentrations were determined using a BCA kit (Thermo Fisher, Cat# 23225). Lysates or purified protein (RBD protein [Acro, Cat# SPD-C52Hh] or Spike-His [described below]) were run on a precast SDS gel (BioRad, Cat# 4561025 and Genscript, Cat# M00652). After transfer, the membrane was stained with Ponceau S solution (Sigma Cat# P3504) to check for total protein transfer. The membrane was then blocked in 5% milk in Tris-buffered saline plus 0.1% Tween 20 (TBST) for 1 hour and put in a primary antibody overnight in 5% bovine serum albumin (BSA) in TBST. The next day, the secondary antibody was diluted in 5% milk in TBST and imaged with an Odyssey fluorescence (LiCor, Odyssey model 9120) or an iBright (Invitrogen, iBright FL1000 Instrument) for chemiluminescence. The following antibodies were used: GFP (Invitrogen, Cat# MA5-15256), CD81 (Abcam, Cat# MA-13548), CD63 (Abcam, Cat# ab59479), actin (Cell Signaling, Cat# 4970), vinculin (Cell Signaling, Cat# 18799), Proteintech 28867-1-AP (raised against amino acids 944–1214 of Wuhan-Hu-1/NC_045512), Novus NB100-56578 directed against the S2 domain (amino acids 1124–1140 of Wuhan-Hu-1/NC_045512), flotillin-2 (Cell Signaling, Cat# 3436), syntenin-1 (Abcam, Cat# ab133267), anti-mouse Licor (Li-Cor, Cat# 926-32212), anti-rabbit Licor (Li-Cor, Cat# 926-68073), anti-rabbit HRP (Vector, Cat# PI-1000), and anti-mouse HRP (Vector, Cat# PI-2000).

### Transmission and cryogenic electron microscopy

For transmission electron microscopy (TEM), total and affinity-purified EVs were adsorbed on glow-charged carbon-coated 400-mesh copper grids for 2 minutes and stained with 2% (wt/vol) uranyl acetate in water. TEM images were taken using a FEI Tecnai 12 transmission electron microscope (Thermo Fisher) at 80 kV. Images were captured on a Gatan Orius camera (2,000 × 2,000 pixels) using the Digital Micrograph software (Gatan, Pleasanton, CA). Zoomed-in images were created and adjusted in Adobe Photoshop (v 2023).

For cryo-EM, samples were absorbed on glow-charged grids (Quantifoil R 1.2/1.3, 400 Mesh, Copper, Cat.Q425CR1.3, EMS) for 30 seconds and blotted for 2 to 4 seconds to remove extra liquid. Then, the grids were snap-frozen in ethane/propane, pre-chilled to −165°C, and imaged using a Thermo Fisher Scientific Talos Arctica G3 instrument. This work was conducted at the UNC CryoEM core.

### Fluorescence microscopy

Cells were seeded onto glass coverslips inside a six-well plate (Fisher 07–200-83) and allowed to grow for 24 hours. Plasmids were then introduced into the cell via transient transfection with Lipofectamine-2000 or 3000 at a 1:2 ratio of DNA to Lipofectamine. The cells were allowed to grow for another 24 hours, fixed with 4% paraformaldehyde in PBS for 15 minutes at room temperature or 100% methanol for 15 minutes at −20°C, and washed with 0.1% TBST. Cells were then blocked with 5% BSA in 0.1% TBST for 30 minutes at room temperature.

Primary antibodies were diluted in 5% goat serum in TBST and incubated with coverslips for 3 hours at room temperature or 4°C overnight. Coverslips were then washed with 0.1% TBST. Secondary antibodies were diluted in 5% goat serum in TBST and incubated with coverslips for 1 hour at room temperature. Cells were washed, and 4′,6-diamidino-2-phenylindole (DAPI) was diluted to a concentration of 0.01% in water and added to the wells for 5 minutes. Coverslips were washed with water and mounted onto Frosted Micro Slides (Corning, Cat# 2948-75x25) using 50 µL of ProLong Gold Antifade Reagent (Cell Signaling 9071S).

Slides were imaged using a DM5500B widefield fluorescence microscope (Leica Microsystems) equipped with a Leica HCX PL apochromatic 100× oil objective with a numerical aperture of 1.40–0.70. Images were captured using a Retiga R3 2.8-megapixel CCD digital microscope camera (Teledyne Photometrics) with a 0.70× magnification c-mount attachment. 2D-deconvolution was then performed on the Z-stacks using MetaMorph 7.8.12.0 software (Molecular Devices). Images were captured using the LAS X software, and deconvolution was performed on the Z-stacks using instant computational clearing. Images were visualized and edited in ImageJ vs 1.8.0_172. Figure S7 slides were taken on an Olympus Fluoview 1,000 confocal microscope using an apochromatic 100× oil objective and captured using the FV1000 software. These images were then visualized and edited using Imaris software version 10.0.

### Super resolution microscopy

Glass-bottom 15 μ-slide eight-well plates (Ibidi Inc., #80827) were prepared by adding 0.01% poly-L-lysine to each well overnight at 4°C. The Cell Mask Red (Thermo Fisher, #C10046) stained EVs were placed into the poly-L-lysine coated wells in a total number of 1 × 10^9^ EVs in 200 µL PBS per well and allowed to adhere to the surface overnight at 4°C. Paraformaldehyde at a concentration of 0.05% in PBS was added to each well and incubated for 30 minutes at room temperature. The solution was then carefully removed with a pipette to avoid disturbing the EVs, and the EVs were washed with PBS. EVs were then blocked with 5% BSA in PBS for 30 minutes at room temperature before antibody labeling as previously described (63, 64).

Antibodies were conjugated to a photo-switchable fluorophore using an Alexa Fluor antibody labeling kit, according to the manufacturer’s protocol. The CD81 antibody (Abcam [M38] ab79559) and S1 Spike antibody (Invitrogen # PA5-114446) were labeled with the Alexa Fluor 488 (Thermo Fisher, Cat# A20181) and Alexa Fluor 568 Labeling Kits (Thermo Fisher, Cat# A20184), respectively. Purified ACE2-His protein (Sino Biological, Cat# 10108-H08H-B) was conjugated using the Alexa Fluor 568 conjugation kit. The first antibody was then diluted in 5% BSA in PBS, and 150 µL was added to each well for 2 hours at room temperature. The antibody solution was removed, and wells were washed with PBS. The blocking and antibody labeling steps were then repeated with the second antibody. B-cubed buffer was then prepared to a 0.05% concentration of enzyme protocatechuate dioxygenase in imaging buffer (ONI, Cat# BCA0017) and added to each well 30 minutes before imaging to scavenge oxidizing molecules.

The Nanoimager S (ONI Inc.) was calibrated for dSTORM using 100 nm Tetraspek microspheres (Invitrogen, Cat# T7279) diluted to 1% in water and placed into Glass Bottom 15 μ-Slide eight-well plates. 3-D mapping calibration and channel mapping calibration were completed to obtain the *x*-, *y*-, and *z*-axis errors. The EVs were then viewed using a custom 405/473/561/640 nm excitation laser configuration (Oxford Nanoimaging). During image acquisition, the laser power was raised by three increments of 10 every 1,000 frames or raised enough to maintain a high signal-to-noise ratio while preventing photobleaching of the fluorescent markers. All experiments were repeated in biological replicates.

During image analysis, post-acquisition correction was performed on the unfiltered image. Photon count, localization precision, sigma, and frame index were adjusted as described in reference 62. Data were then analyzed using the CODI program (Oxford Nanoimaging). Colocalization data were exported to Microsoft Excel, and pie charts were made in R 4.2.1 using the ggplot2 package.

### Animal studies

Mice were maintained and bred in a pathogen-free animal biosafety level 1 (ABSL1) facility under the care of the UNC Division of Comparative Medicine and UNC Animal Studies Core (ASC). Experimental manipulations occurred in an ABSL2 facility, where mice were housed under aseptic conditions.

### Administration of EVs in mice

EVs were thawed to room temperature (RT) without external heat inside a biosafety cabinet to maintain sterility. Injection preparations were made by diluting EVs to 1 × 10^10^ particles/mL in sterile PBS. EVs were thoroughly mixed in the biosafety cabinet by pipette.

The EVs were prepared on the same day and given to the ASC for subcutaneous injection at a volume of 100 µL per animal. Mice were divided into two treatment groups: EV or PBS injection. There were three injections given in total, 14 days apart. Seven days after the final injection, the mice were sacrificed for sample collection. Whole blood was collected via cardiac puncture, and spleens were harvested and fixed in 10% buffered formalin for 24 hours at 4°C.

### Serum analysis

The whole blood was collected into untreated sterile microcentrifuge tubes and was allowed to clot upright for 30 minutes at room temperature. The serum was then separated by centrifugation at 4°C and 7,500 × g for 15 minutes. The serum was collected, diluted 1:1 with PBS, and incubated in a 56°C dry bath for 30 minutes to inactivate complement. Antibody levels were determined using a Mouse Anti-SARS-CoV-2 IgG Titer Serologic Assay Kit (Spike trimer) from Acro Biosystems (Cat# RAS-T023). The enzyme-linked immunosorbent assay (ELISA) was performed according to the manufacturer’s instructions using a final serum dilution of 1:1,000. Positive and negative controls were similarly diluted at 1:1,000. Mouse anti-SARS-CoV-2 IgG standards provided by the manufacturer were serially diluted to create a standard curve for quantification.

Samples for the ELISA were plated in duplicate, and washes were performed using a BioTek ELx405 microplate washer. Absorbance was detected as optical density using a CLARIOstar Plus microplate reader (BMG Labtech) at 450 nm with 630 nm as the reference wavelength. Titers for serum samples were determined by applying the appropriate reference and blank corrections and then plotting the duplicate average on the standard curve to obtain the calculated sample concentration. Multiplying by the dilution factor yielded the serum antibody concentration in ng/mL.

### Purification of Spike-His

The secreted form of stabilized Spike protein expression plasmid (pDD3503) was a gift from Dr. Florian Krammer (61). The Spike-His construct was transfected into HEK293T cells. After 48 hours, the media was harvested by spinning at 1,000 × g for 10 minutes and then filtered through a 0.45 µm filter. Protease inhibitor (Thermo Scientific 78429) was added to the filtered media to prevent Spike degradation, and 20 mL of 20 mM sodium phosphate, 10 mM imidazole, 300 mM sodium chloride, pH 7.4 were mixed into the media. The media were then run through a HisTrap HP column (Cytiva, Cat# 29051021) for affinity purification by the histidine tag of the Spike protein. The column was washed with 20 mM sodium phosphate, 25 mM imidazole, 300 mM sodium chloride, pH 7.4. The Spike-his construct was then eluted with an elution buffer of 20 mM sodium phosphate, 500 mM imidazole, 300 mM sodium chloride, pH 7.4. The Spike-His was then kept at −80°C until use.

### Flow cytometry and cell sorting

BD Accuri C6 Plus Flow Cytometer was calibrated with two drops of CS&T RUO beads diluted in 500 µL nanoparticle water (BD Biosciences 661414). Gates were created to select singlets and live cells based on FSC-A × FSC-H and FSC-A × SSC-A. Upon confluency, CD81-Strep-GFP, CD81-Strep-S-GFP, and U-2 OS WT cells were rinsed with Dulbecco’s phosphate-buffered saline (Gibco, 14190-144) of equivalent media volume, followed by incubation with 0.05% trypsin-EDTA (Gibco, Thermo Fisher, P: 25300-054) for 5 min at 37°C. One million cells were used for reading by flow cytometer. CD81-Strep-GFP or CD81-Strep-Spike-GFP cells were reported through GFP fluorescence using a FITC optical filter. U-2 OS WT cells were used as control. Clones with a single peak of FITC-H+ count were selected to establish permanent cell lines. Each selected colony was read three times over 3 weeks to ensure that the transfected plasmids were maintained.

## RESULTS

### Characterization of CD81-S fusion proteins

Past studies suggested that CD81 can direct the intracellular trafficking of molecules (50). Therefore, we fused S into amino acids 179 to 180 (based on UniProt ID: P60033) of CD81. This region of CD81 is not resolved in the crystal structure. It is considered flexible and solvent exposed (48, 51). Figure 1 summarizes the constructs used. ^D614G^S_WA1_::GFP (MN908947.3, strain Wash1) served as wild-type control for the native trafficking of S. CD81::GFP::^D614G^S_WA1_ and CD81::GFP::^D614G^S_WA1-FCI_ are CD81 fusions with either an intact or mutated furin cleavage site. As an additional control, we used a secreted form of S (61). This variant contains two proline mutations at 986 and 987 [called S(P2)], has the furin cleave site deleted, and is codon optimized but does not contain the D614G mutation. In addition, it contains the trimerization domain (foldon) of T4 fibritin21 fused to its C-terminus for multimerization and a 6xHis tag for purification (6, 61, 65). It is henceforth referred to as S_WA1_::HIS.

We also explored the high morbidity Delta (B.1.617.2) strain. In strain Delta, the S protein has the following substitutions in addition to D614G: T19R, G142D, R158G, L452R, T478K, P681, and D950N, as well as deletions of E156 and F157. Previous research found that the Delta strain of SARS-CoV-2 has higher infectivity compared to other strains, possibly due to the higher binding efficiency with the ACE2 receptor (66). We used an S backbone that also contained HexaPro stabilizing mutations and had the furin cleavage site deleted (24). We hypothesized that removal of the furin cleavage site might decrease degradation. It represents the S molecules used in current vaccines. To explore the impact of the furin site, we created an isogenic derivative with or without furin/polybasic site reconstituted: CD81::GFP::^D614G^S_delta_ and CD81::GFP::^D614G^S_delta-FCI_.

**Fig 2:**
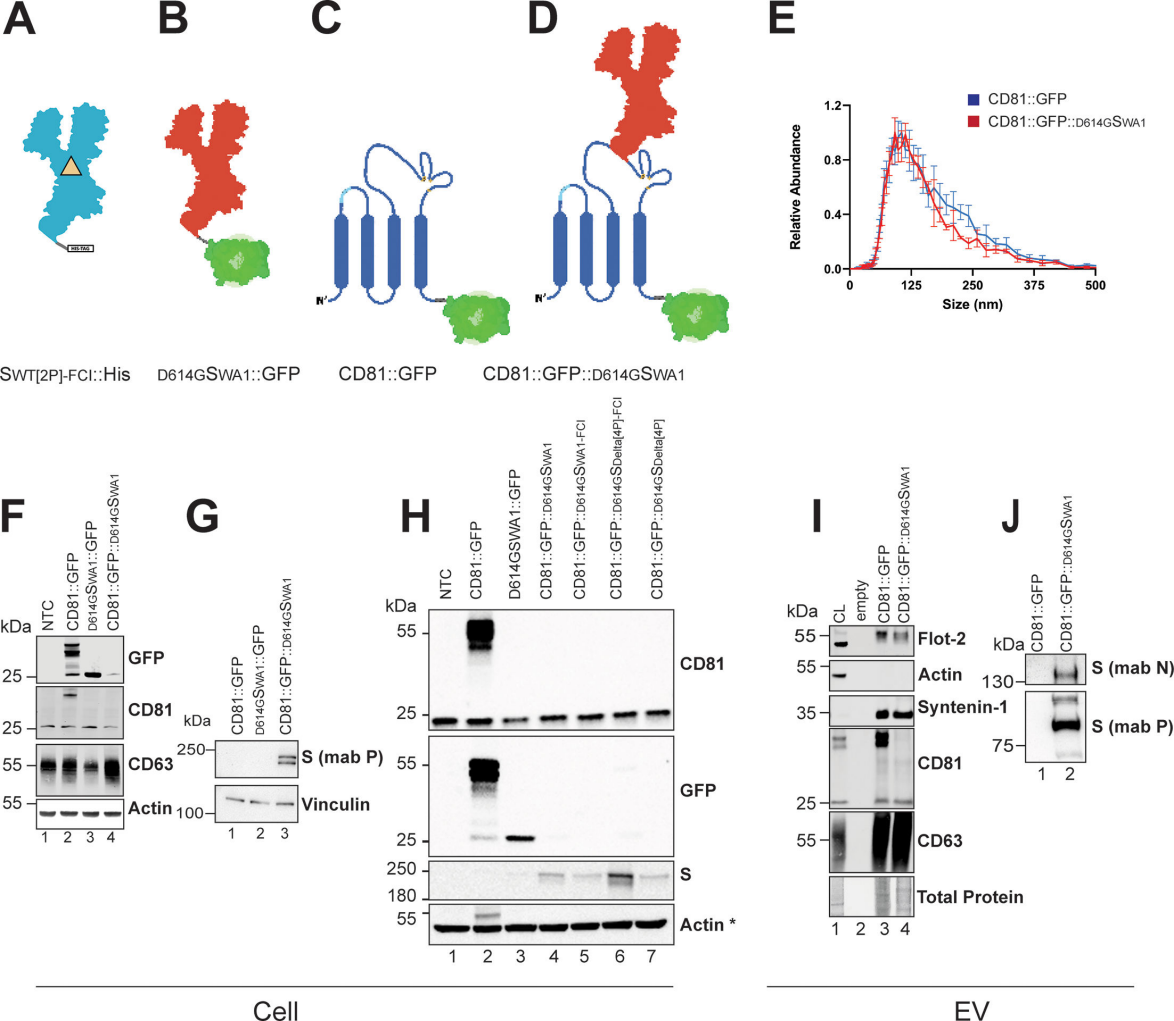
Expression of recombinant recombinant CD81::S constructs. (**A–D**) Pictographs of constructs are used in this research. (**A**) A stabilized spike-his construct with the FCI removed as indicated by the yellow triangle within the turquoise outline and a bacterial trimerization domain: S_WT[2P]-FCI_::His. (**B**) Wild-type Spike strain WA1 with the D614G SNV containing the FCI is in red with GFP (in green) on the C-terminus: ^D614G^S_WA1_::GFP. (**C**) CD81 molecule (in blue) with GFP on the C-terminus: CD81::GFP. (**D**) Non-stabilized Spike strain WA1 with the D614G SNV containing the FCI (in red) cloned into the large extracellular loop of CD81 with GFP on the C-terminus: CD81::GFP::^D614G^S_WA1_. (**E**) EVs size and concentration after purification from cells stably expressing CD81::GFP or CD81::GFP::^D614G^S_WA1_. EVs were then diluted in water and analyzed using the ZetaView. The size and concentration of the particles were measured with three reads per experiment and three separate experiments. (**F–H**) Western blot analysis of whole cell lysates. (**F**) Cell lysates from U-2 OS cells stably express the constructs. (**G**) HEK293T cells were transfected with CD81::GFP, ^D614G^S_WA1_::GFP, or CD81::GFP::^D614G^S_WA1_. Then, the cells were harvested, lysed, and probed against Spike and the loading control vinculin. (**H**) HEK293T cells were transfected with CD81::GFP ^D614G^S_WA1_::GFP, and the constructs were described in Table S1. The lysates were then run on an SDS-PAGE gel and probed with the indicated antibodies. NTC stands for no transfection control. (**I–J**) Western blot analysis of EV lysate. (**I**) Analysis of EV protein enrichment. Cell lysate was used as a control. EVs were harvested from U-2 OS cells stably expressing CD81::GFP or CD81::GFP::^D614G^S_WA1_. These EVs are enriched in proteins important for biogenesis, including CD63 and Syntenin-1, and do not have the cellular protein actin. (**J**) EVs purified from these cell lines were lysed and run on an SDS-PAGE gel. Two anti-S antibodies (mab N and mab P) were used to show S expression in the lysate. All experiments were conducted in at ≥3 biological replicates; * indicates a reprobed blot (hence the upper band in lane 2 representing CD81-GFP).

All constructs expressed full-length fusion proteins upon transient transfection of HEK293T cells. Figure 2H shows a comparative western blot of whole cell lysate for all constructs. There were some technical peculiarities due to the specificity of the available antibodies and the large difference in size. First, not all anti-S antibodies recognize all SARS-CoV-2 lineages with similar sensitivity and specificity. Second, CD81 has an apparent molecular weight of 27 kDa, while full-length (S0) S has an apparent molecular weight of 180 kDa or higher depending on mutation composition, presence of the furin cleavage site, and glycosylation (37). No one gel can resolve both species. Additionally, CD81 cannot be resolved in the CD81::S::GFP constructs since S is inserted into the same domain that is recognized by anti-CD81 antibodies. Interestingly, we found that the deletion of the furin cleavage insert (FCI) in the cells expressing CD81::GFP::^D614G^S_delta_ has a higher expression of S protein than when the FCI is intact.

We used the C-terminal GFP tag (Fig. S1) as readouts in FACS and selection in G418 to derive stable cell U-2 OS clones that express the recombinant CD81::GFP::^D614G^S_WA1_ fusion protein (cell lines U-2 OS CD81::GPF or U-2 OS CD81::GFP::S_WA1_). We used U-2 OS cells as these are larger and thus better suited for subsequent protein localization studies than HEK293T cells. Western blot validated the expression for these stable cell lines (U-2 OS) and transiently transfected controls (HEK293T; Fig. 2). All cells expressed endogenous CD81 at ~27 kD apparent molecular weight, detected by anti-CD81 antibody. The CD81::GFP transfected cell lines showed two bands, the endogenous CD81 at ~27 kD and the CD81::GFP fusion protein at ~50 kD. The WT cells were, expectedly, negative for GFP, which also has an apparent molecular weight of ~27 kD and is detectable with anti-GFP antibody. The ^D614G^S_WA1_::GFP clone showed a prominent GFP band ~25 kDa but no GFP reactivity at higher molecular weight. This was similar to the result after transient expression in HEK293T cells (Fig. 2H). We surmise that all or a fraction of GFP is cleaved off. No “free” GFP was seen in the CD81::GFP:: ^D614G^S_WA1_ cell line, where GFP was fused to the C-terminus of the CD81, not to S directly. Actin (42 kDa apparent MW) was used as a loading control in each instance. Also shown is another tetraspanin, CD63, which is highly glycosylated and migrates at an apparent molecular weight of ~55 kDa. Based on probing for the C-terminal GFP tag, the stable cell lines expressed the recombinant proteins. S was not detectable in the whole cell extract ^D614G^S_WA1_::GFP or mock-transfected U-2 OS cells (Fig. 2F). This is consistent with our experience that S overexpression in isolation is toxic to cells unless redirected into the secretory pathway, as in S_WA1_::HIS. Alternatively, the ^D614G^S_WA1_ variant, in the absence of NS orf3a, is being directed into the lysosome and degraded in the absence of virion formation (30). Placing ^D614G^S_WA1_ within the LEL of CD81::GFP rescued expression of S. Using an anti-S2 antibody, two bands around ~180 kDa were detectable in whole cell lysates of cells containing the full length, or S0, CD81::GFP:: ^D614G^S_WA1_ -GFP expression construct. Vinculin (116–130 kDa apparent MW) was used as a loading control.

### CD81 fusion alters the intracellular trafficking of ^D614G^S (WA1)

Next, we tested the hypothesis that a CD81::GFP:: ^D614G^S_WA1_ fusion would direct S into EV by means of particle purification. EVs were purified from conditioned cell media from U-2 OS cells stable expressing CD81::GFP or CD81::GFP:: ^D614G^S_WA1_ using our validated four-step purification pipeline (67). The EVs were positive for known EV markers such as Syntenin-1 and CD63 but not for cellular actin (Fig. 2I). EV purified from CD81::GFP:: ^D614G^S_WA1_ producer cells contained the S protein (Fig. 2J) as determined by using two antibodies. NTA found no difference in EVs’ number or size distribution (Fig. 2E). The NTA size measurements were confirmed by TEM (see Fig. 4 below). Fusing S to CD81 rescued ^D614G^S_WA1_ from degradation and directed it into EVs.

**Fig 3:**
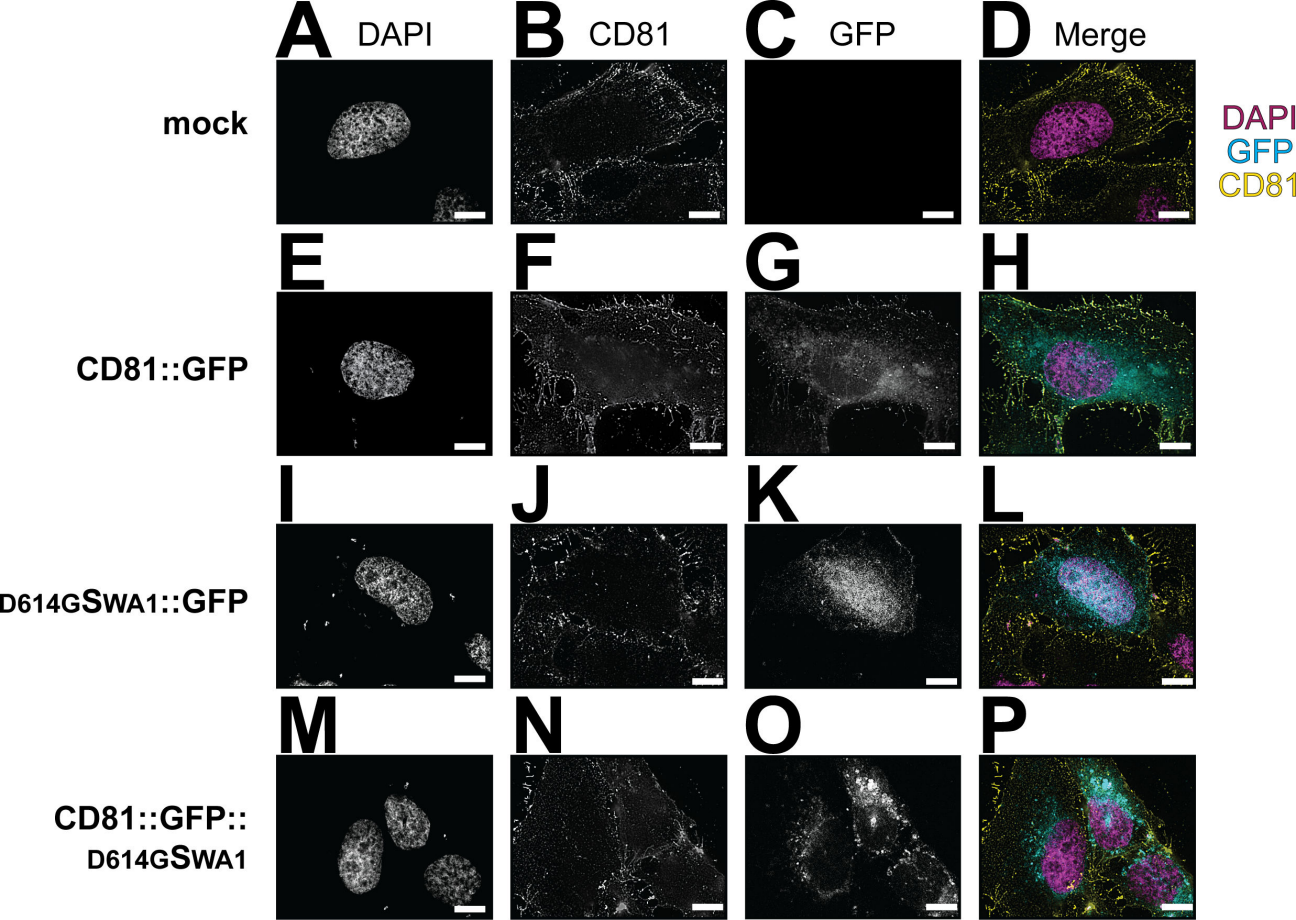
CD81::S fusion protein has altered localization. Cells were seeded on coverslips and transfected with CD81::GFP, ^D614G^S_WA1_::GFP, or CD81::GFP::^D614G^S_WA1_, in addition to a non-transfected control. Twenty-four hours post-transfection, the cells were fixed with methanol and stained with a CD81 antibody. (**A–D**) Cells with no DNA added. (**E–H**) Cells transfected with CD81::GFP. The GFP signal is diffused through the cell and on the plasma membrane. (**I–L**) Cells transfected with ^D614G^S_WA1_::GFP. The GFP signal is located around the nucleus. (**M–P**) Cells transfected with CD81::GFP::^D614G^S_WA1_. The GFP signal is diffused through the cell. The CD81 antibody does not bind to this construct since the Spike covers the binding area. All experiments were conducted in at ≥3 biological replicates. The yellow arrow highlights different vesicle localization of^D614G^S_WA1_::GFP as compared to CD81::GFP::^D614G^S_WA1_. Scale bar = 10 µm.

To confirm the biochemical results by an orthogonal approach, we utilized deconvolution-enhanced fluorescence microscopy (Fig. 3) on permeabilized cells. U-2 OS cells were transfected with the different recombinant CD81 plasmids as before. Twenty-four hours post-transfection, cells were fixed and stained with an anti-CD81 antibody (clone MA5). The nuclei were visualized with DAPI. In the non-transfected cells, there was no GFP signal. Endogenous CD81 was detectable on the plasma membrane as expected (Fig. 3). Upon transfection, the CD81::GFP fusion molecule colocalized with total CD81 at the cell surface (Fig. 3E through H). The C-terminal, internal GFP-tag did not alter CD81 localization. The CD81 trafficking signals were dominant over the GFP trafficking signals. The ^D614G^S_WA1_::GFP was poorly expressed, with the GFP signal around the nucleus (Fig. 3I through L). This observation matches the western blot data in Fig. 2. It is consistent with the interpretation that GFP was cleaved and that ^D614G^S_WA1_ does not express well or is toxic to cells. Endogenous CD81 localization was not affected by ^D614G^S_WA1_::GFP transfection. The localization of CD81::GFP:: ^D614G^S_WA1_ matched that of CD81::GFP (Fig. 3G, D, and M through P).

**Fig 4:**
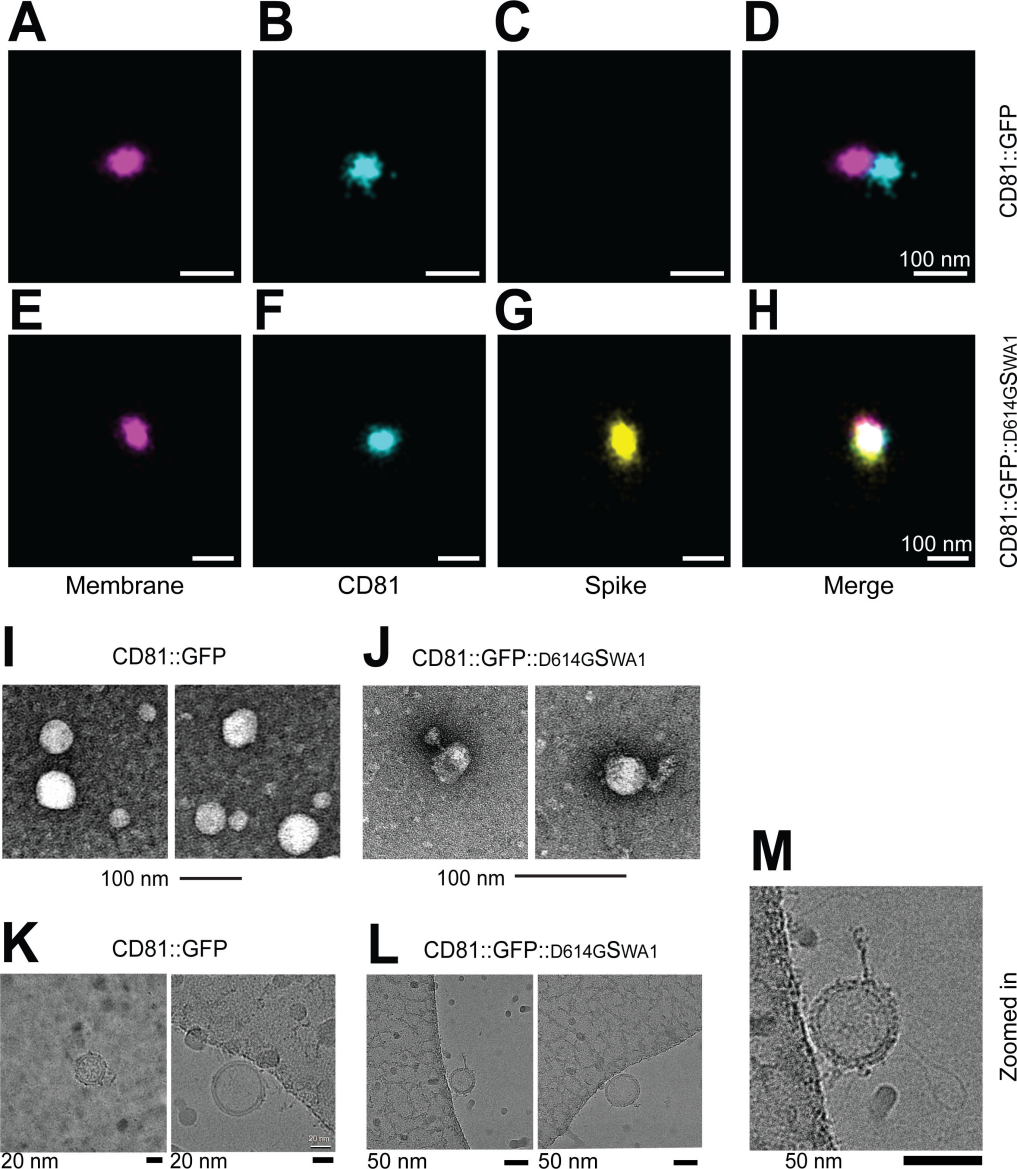
Single particle analysis using dSTORM, TEM, and CryoEM. EVs harvested from cells expressing CD81::GFP or CD81::GFP::^D614G^S_WA1_ were analyzed using different techniques. First, EVs were seeded onto Ibidi 8-well glass bottom chamber slides. The EVs were stained with a CD81-Alexa488 antibody and then a S-Alexa594 antibody. After staining, the EVs were washed and placed in an oxygen scavenging buffer (B^3^; ONI) and imaged using the Nanoimager from Oxford Nanoimaging. Example images are taken from EVs the backbone only CD81::GFP EVs (**A–D**) or the CD81::GFP::^D614G^S_WA1_ EVs (**E–H**). Next, EVs were stained using uranyl acetate seeded onto a carbon-coated copper grid and imaged using TEM. (**I**) CD81::GFP EVs and (**J**) CD81::GFP::^D614G^S_WA1_ EVs images taken using TEM. Finally, EV samples were absorbed onto a copper grid, then snap-frozen in ethane/propane to be imaged using cryo-EM. (**K**) CD81::GFP EVs and (**L**) CD81::GFP::^D614G^S_WA1_ images taken using cryo-EM. All experiments were conducted in at ≥3 biological replicates.

To refine our analyses, we used single-particle visualization and quantitation. We previously validated dSTORM as a reproducible and robust assay to determine the presence and localization of proteins on individual EVs (64). Here, purified EVs are marked by a membrane stain to distinguish them from protein aggregates (pseudo-colored magenta in Fig. 4). To detect colocalization on an individual EV, the particles were incubated with directly conjugated anti-S1 (clone XPA5, ALEXA-594, pseudo-colored yellow) or anti-CD81 (clone M38, ALEXA-488, pseudo-colored cyan) antibodies, followed by washing and visualization. Many EVs purified from CD81::GFP transfected cells reacted with the CD81 antibody (Fig. 4A through D). Some EV purified from CD81::GFP:: ^D614G^S_WA1_ transfected cells reacted with anti-CD81 and anti-S antibodies (Fig. 4E through H). This confirms the presence of CD81::GFP:: ^D614G^S_WA1_ in highly purified EV, independently of the GFP tag. Because S is an extended molecule (~ 20 nm head-to-membrane domain [21, 23]), we were able to visualize the protein directly on the 60–120 nm vesicles. Under TEM, CD81::GFP and CD81::GFP:: ^D614G^S_WA1_ EVs were of similar size (Fig. 4I and J). Unlike in the CD81::GFP EV preparation, the CD81::GFP:: ^D614G^S_WA1_ EV contained many particles with a hammer-shaped protrusion, indicative of S loading. This observation was confirmed using cryo-EM. As in the TEM experiment, the CD81::GFP EVs were round with only small protein protrusions. By contrast, EV purified from CD81::GFP:: ^D614G^S_WA1_ transfected cells had a large, extended, and hook-like structure emanating from their surface (Fig. 4K and L).

### CD81 fusion of a stabilized ^D614G^S (Delta) results in high-efficiency EV loading

**Fig 5:**
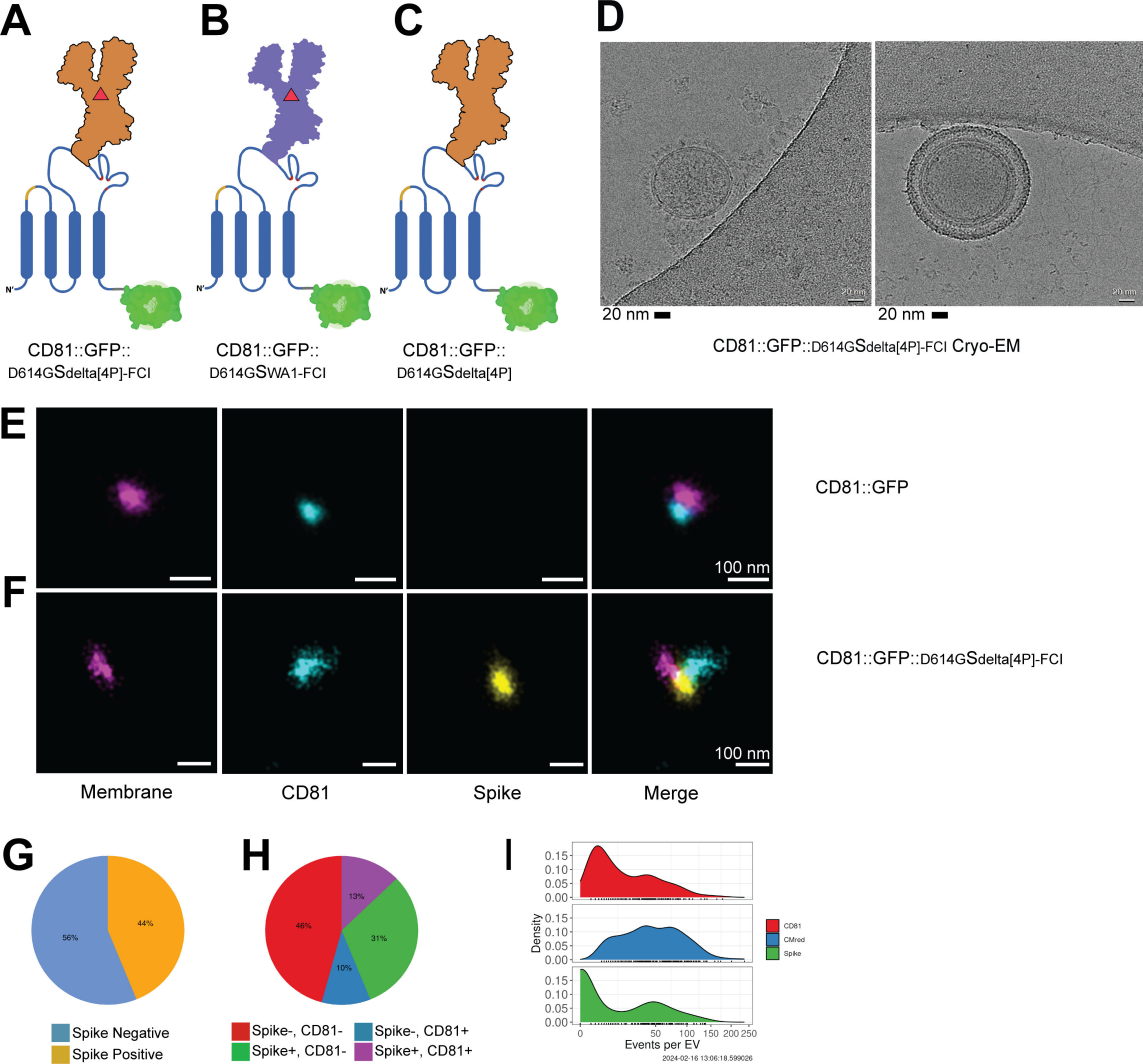
Stabilization of S increases the expression levels on EV. (**A**) CD81-Delta Spike-GFP with hexa-proline mutations and the FCI removed, CD81::GFP::^D614G^S_delta[4P]-FCI_, the primary construct used in the rest of the paper. (**B**) CD81::GFP::^D614G^S_WA1-FCI_ with the FCI removed. (**C**) CD81::GFP::^D614G^S_delta[4P]_ with hexa-proline mutations but with the FCI intact. (**D**) Example images of cryo-EM images. EV samples were absorbed onto a copper grid, then snap-frozen in ethane/propane to be imaged using cryo-EM. (**E–I**) First, EVs were seeded onto Ibidi 8-well glass bottom chamber slides. The EVs were stained with a CD81-488 antibody and then a Spike-594 antibody. After staining, the EVs were washed and placed in an oxygen scavenging buffer (B^3^; ONI) and imaged using the Nanoimager from ONI. Representative dSTORM images taken from EVs, the backbone only CD81::GFP EVs (**E**) or the stabilized CD81::GFP::^D614G^S_delta[4P]-FCI_ EVs (**F**). During image acquisition, at least 2,000 EVs taken from three separate frames were analyzed using ONI CODI software. The Excel reports were analyzed in R to create pie charts to determine the percent of EVs carrying S (**G**) or the percent of EVs positive for CD81, S, or both. (**H and I**) Similar to G, the amount of EVs positive for CD81 or S. All experiments were conducted in at ≥3 biological replicates.

Although the result using a native CD81::GFP:: ^D614G^S_WA1_ fusion confirmed the overall hypothesis, the loading efficiency into EV was not very high. The SARS-CoV-2 Delta variant (PANGO lineage B.1.617.2) is associated with better infectivity in ACE2-expressing cells, a more drastic phenotype in experimental models, and more severe clinical symptoms (68). We, therefore, repeated the experiment using proline-stabilized ^D614G^S_delta[4P]_ variants. Figure 5A through C summarizes the design. To investigate the importance of the furin/polybasic cleavage site, isogenic recombinants with either wild type or inactive furin site were created in both the wild-type ^D614G^S_WA1_ and the ^D614G^S_delta[4P]_ fusion. The state of the furin site cleave site did not affect the trafficking of the CD81::GFP::S fusion inside the cell (Fig. S2). EVs purified from cells transfected with CD81::GFP reacted with anti-CD81 antibody only (Fig. 5E). In contrast, EVs purified from cells transfected with CD81::GFP:: ^D614G^S_delta[4P]_ reacted with both anti-CD81 and anti-S monoclonal antibodies (Fig. 5F). Two thousand four hundred ninety-eight events were quantitated, counting only those signals that colocalized with an EV membrane signal. Of all EVs stained with anti-S antibody, 44% were stained (Fig. 5G), and 13% were double positive for S and CD81 (Fig. 5H). The level of signal events on any individual EV was similar to that of CD81 (Fig. 5I). For the first time, more than one S protrusion decorated the EV (Fig. 5D). Some EVs were decorated by an aura of S molecules, exceeding the packaging density of the native virus.

Next, we studied the subcellular localization of the CD81::GFP:: ^D614G^S_delta[4P]_ molecule. We imaged CD63, which accumulates in the MVB before being extruded as part of EV (69) and LAMP1 as a lysosome marker (Fig. S3). The cells were transfected with either ^D614G^S_WA1_::GFP, CD81::GFP, or CD81::GFP:: ^D614G^S_delta[4P]_, and imaged at 6, 12, and 24 hours after transfection (Fig. S4). ^D614G^S_WA1_::GFP accumulated in the nucleus (as in Fig. 3), while CD81::GFP mimicked the staining pattern of endogenous CD81 (as in Fig. 2). At 12 hours, CD81::GFP and CD81::GFP:: ^D614G^S_delta[4P]_ were moving toward the plasma membrane (Fig. S4M through R), while ^D614G^S_WA1_::GFP was mostly near the nucleus (Fig. S4J through L). At 24 hours, transfected CD81::GFP had accumulated at the cell membrane, overlapping the endogenous CD81 signal (Fig. S4V through X), while ^D614G^S_WA1_::GFP remained around the nucleus (Fig. S4S through U), and CD81::GFP:: ^D614G^S_delta[4P]_ was detectable both within the cytoplasm and on the cell membrane (Fig. S4Y through AA). These experiments were repeated using CD63 (Fig. S5) or LAMP1 (Fig. S6) as the cellular marker with similar results. ^D614G^S_WA1_::GFP localization correlated with the LAMP1 signal, as previously described (19), but the CD81::GFP:: ^D614G^S_delta[4P]_ did not.

As described previously, the accessory protein orf3a aids in Spike trafficking during infection (30). We transfected U-2 OS cells with orf3a::mCherry, ^D614G^S_WA1_::GFP, CD81::GFP:: ^D614G^S_delta[4P]_, or a combination (Fig. S7). We found that orf3a causes punctate localization when expressed alone (Fig. S7A). Similar to our other images,^D614G^S_WA1_::GFP was close to the nucleus (Fig. S7B), while CD81::GFP:: ^D614G^S_delta[4P]_ is found throughout the cell (Fig. S7C). When we co-transfect orf3a::mCherry with CD81::GFP, we see a similar puncta of orf3a, with no change to CD81::GFPs localization on the membrane (Fig. S7D). However, when cells expressing both orf3a::mCherry and ^D614G^S_WA1_::GFP, the proteins are highly co-localized together, in addition to localizing with the lysosome marker LAMP1, as previously described (30). Interestingly, when orf3a::mCherry is expressed with CD81::GFP:: ^D614G^S_delta[4P]_, the CD81::GFP:: ^D614G^S_delta[4P]_ becomes more internalized and looks more similar to ^D614G^S_WA1_::GFP rather than CD81::GFP. This leads us to hypothesize that orf3a has a stronger trafficking effect compared to CD81. In conclusion, fusion to the tetraspanin CD81 directed stabilized S into the CD81 biogenesis pathway and with high efficiency into EVs.

### EV carrying the CD81:^D614G^S (Delta) fusion protein bind ACE2

**Fig 6:**
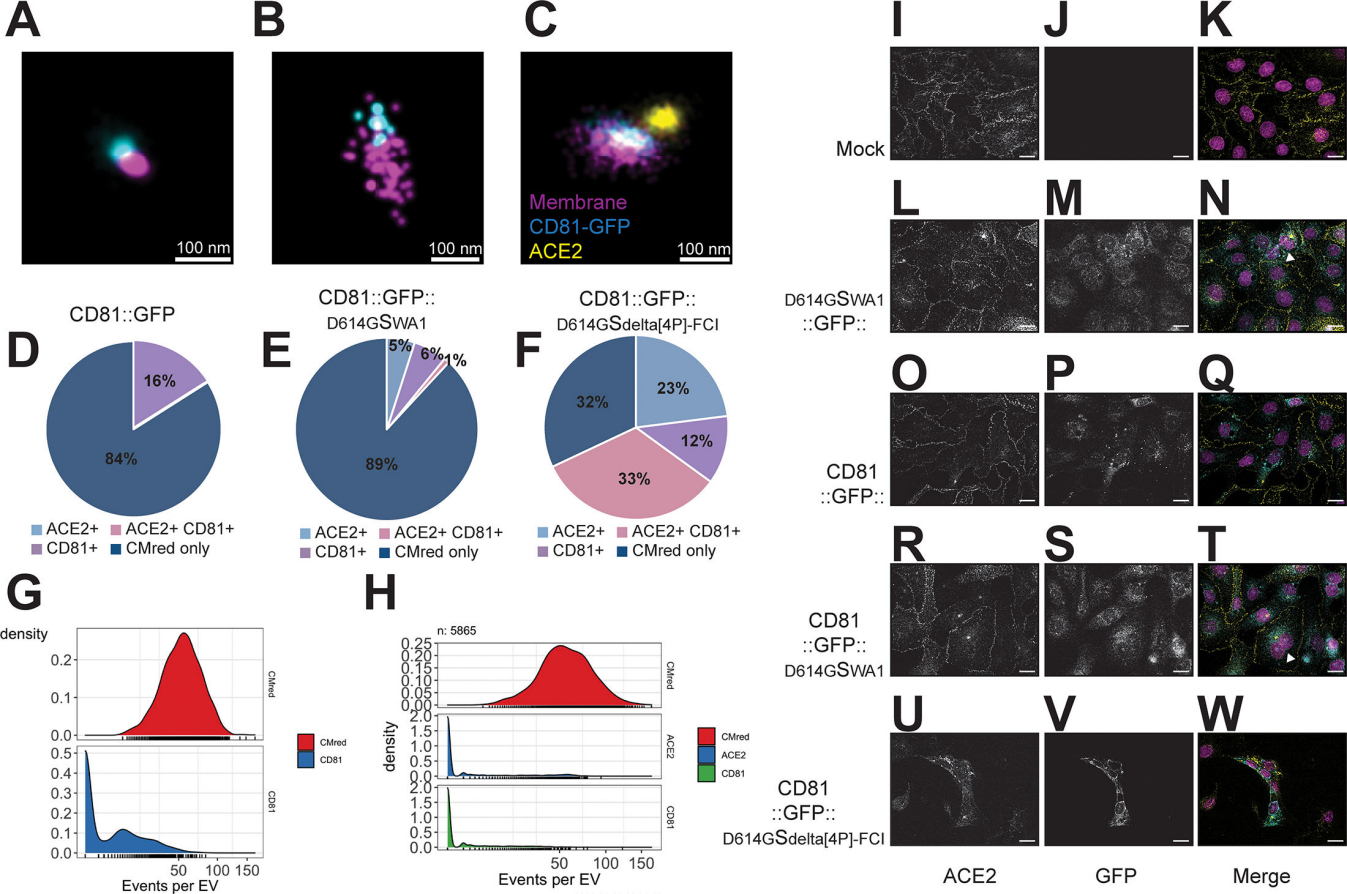
CD81::S EV bind to ACE2. EVs from (**A**) CD81::GFP, (**B**) CD81::GFP::^D614G^S_WA1-FCI_, or (**C**) CD81::GFP::^D614G^S_delta[4P]-FCI_ were seeded onto Ibidi 8-well glass bottom chamber slides. The EVs were then incubated with purified ACE2 (Sino Biological, Cat# 10108-H08H-B) conjugated to Alexa fluor 594. After staining, the EVs were washed and placed in an oxygen scavenging buffer (B3; ONI) and imaged using the Nanoimager from Oxford Nanoimaging. During image acquisition, at least 2,000 EVs taken from three separate frames were analyzed using ONI CODI software. The Excel reports were analyzed in R to create pie charts to determine the percent of EVs that bound to ACE2 or had CD81-GFP signal (**D–F**). Similar to panels D and F, the amount of EVs positive for CD81 or Spike is graphed in panel R (**G and H**). Vero cells overexpressing human ACE2 were transfected with (**I–K**) no DNA, (**L–N**) ^D614G^Spike_WA1_::GFP, (**O–Q**) CD81::GFP, (**R–T**) CD81::GFP::^D614G^S_WA1_, or (**U–W**) CD81::GFP::^D614G^S_WA1-FCI_. Previous research showed that co-expression of S and ACE2 induces syncytia formation. Scale bar = 10 µm.

To test the hypothesis that CD81::GFP:: ^D614G^S_delta[4P]_ assembled into a correctly folded trimer on an EV, we evaluated ACE2 binding. ACE2 conjugated to an AlexaFluor-568 antibody, which is pseudo-colored yellow, was incubated with S-EVs and imaged by total internal reflection fluorescence (TIRF) microscopy. CD81::GFP EV (Fig. 6A) did not bind ACE2. EVs from cells expressing CD81::GFP:: ^D614G^S_WA1_ (Fig. 6B) bound ACE2, but at a low percentage (6%) and EVs expressing CD81::GFP:: ^D614G^S_delta[4P]_ bound ACE2 at a high percentage (56%) (Fig. 6C).

S can induce syncytia formation in cells expressing ACE2 (70). To further validate our constructs, we repeated these findings by transfecting the CD81::S fusion constructs into Vero cells expressing human ACE2 (Fig. 6I through W). Wild type ^D614G^S_WA1_::GFP induced cell fusion (Fig. 6L through N, white arrow). CD81::GFP:: ^D614G^S_WA1_ also induced cell fusion (Fig. 6R through T, white arrows) but CD81::GFP did not. Pastorio et al. found that the Delta S induced more cell fusion events when compared to other S mutants (70). This observation held true. The CD81::GFP:: ^D614G^S_delta[4P]_ induced more cell fusion than CD81::GFP:: ^D614G^S_WA1_ (Fig. 6U through W). These data show that the CD81::GFP::S molecules form a complex with ACE2 on the cell surface and on EV.

### EVs carrying the recombinant CD81:^D614G^S (Delta) protein induce anti-RBD antibodies

**Fig 7:**
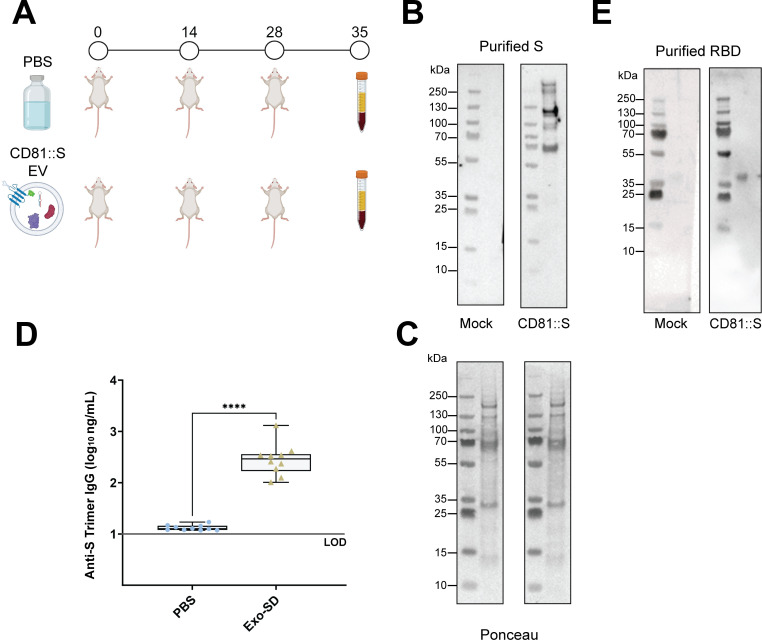
Stabilized CD81::GFP::^D614G^S_delta[4P]-FCI_ EV induce S-Trimer and RBD antibodies. (**A**) Illustration of the treatment plan and boost regimen. The numbers on top indicate days of injection or (day 35) collection. The group size was *n* = 10. (**B**) Western blot assay to test sera for the presence of anti-S antibodies. The target S_WT[2P]-FCI_::His was collected from conditioned cell media and purified using a His column. Protein was run on an SDS-PAGE gel. Serum from mice injected with PBS or EV-CD81::GFP::^D614G^S_delta[4P]-FCI_ was incubated with the membranes at 1:100 dilution and detected with anti-murine total IgG-conjugated to horseradish peroxidase. Weak bands on the left and numbers indicate molecular weight markers in kDa. (**C**) Results of an ELISA assay testing for the presence of S-trimer-specific antibodies. This uses a commercial ELISA (Acro Biosystems RAS-T023). Shown is a box and whisker plot of the range, median, first and third quartile overlayed with individual data points for either the S negative group (blue) or the S positive group (brown). The amount of anti-S-specific IgG is shown on the vertical axis in ng/mL on a log 10 scale. Significance is indicated by the number of stars with ****: *P* ≤ 0.0001 by one-way analysis of variance with multiple comparisons. (**D**) Western blot assay to test sera for the presence of RBD antibodies. Purified RBD protein (ACRO) was run on an SDS-PAGE gel. Serum from mice injected with PBS or EV-S was incubated with the membranes at 1:100 dilution and detected with anti-murine total IgG-conjugated to horseradish peroxidase. Weak bands on the left and numbers indicate molecular weight markers in kDa.

Lastly, we explored if CD81::GFP:: ^D614G^S_delta[4P]_ EVs could induce anti-S antibodies. Ten mice each were injected with 1 × 10^9^ EV containing CD81::GFP:: ^D614G^S_delta[4P]_ (Exo-Spike) in sterile PBS or a PBS control three times, 2 weeks apart. The mice were monitored for signs of toxicity; none experienced any (Fig. S9). One week after the last injection, serum was harvested, and the presence of anti-S antibodies was determined. Western blotting serum from Exo-Spike treated mice reacted with purified S_WT[2P]-FCI_::His, whereas serum from mock-treated mice did not (Fig. 7A through C). An anti-S IgG ELISA serum from Exo-Spike animals had significantly higher reactivity than serum from mock-treated animals (Fig. 7D). To determine if Exo-Spike particles produced antibodies against the RBD domain, we used purified Delta Spike RBD (ACRO Cat#SPD-C52Hh) as the target in a western blot assay. Serum from mice treated with Exo-Spike recognized RBD, but the mock serum did not. To determine the level of batch variation, we repeated the experiment. The second Exo-Spike batch produced antibodies similar to the first batch (Fig. S8).

## DISCUSSION

Current SARS-CoV-2 mRNA vaccines contain the stabilized S as their sole antigen. This is in contrast to natural infection, during which E, M, and nonstructural proteins are co-expressed. Some nonstructural viral proteins, such as orf3A, have known functions in S protein biosynthesis (30, 71). The biogenesis pathway of S delivered by mRNA vaccination does not resemble the biogenesis pathway of S in the context of natural infection. The impact of this difference in S biogenesis is not known. This is the knowledge gap that this study tried to fill.

Grafting S onto the tetraspanin CD81 altered S-intracellular trafficking, alleviated S-induced cellular toxicity, and delivered a large, intact, and correctly folded fraction of S into EVs. The CD81 trafficking signals overrode the native S trafficking signals. Presumably, the non-covalent association of S1 and S2 anchored the CD81 four transmembrane bundle and kept the overall complex intact, analogous to the trimerization domain of T4 fibritin21 used in secreted S proteins (24, 61). The EV-based S nanoparticles bound ACE2, evidencing solvent exposure of the correctly folded RBD, and displayed multiple S copies per particle analogous to the native virion. These nanoparticles are immunogenic in mice in the absence of any adjuvant.

TSPANs can be divided into five largely independent parts: an amino-terminal intracellular domain, the four-helix transmembrane bundle, the SEL, the LEL, and the carboxyl-terminal domain. The four-helix transmembrane bundle holds these different domains. The TSPAN LEL contains a conserved helix region and a variable domain containing Cys residues, which form two to four disulfide bridges (72). CD81, in particular, has a two-loop structure stapled together by two disulfide bridges, which are conserved across different TSPANs and across the animal kingdom (73, 74). Maintaining the disulfide bridges in the LEL contributes to CD81 stability (75–77). Hence, engineering functional TSPAN fusion proteins is not obvious. CD81 stands out among the TSPAN for two reasons. Historically, it was identified as the HCV receptor in liver cells (58, 59, 78). It heterodimerizes with CD19 on B cells; some argue that CD81 dynamically competes with the B cell receptor (BCR) for CD19 binding (50). Therefore, we suspect that the CD81 LEL is more flexible than other TSPANs despite carrying the canonical cysteine quadruplet. Unlike other TSPAN, the CD81 targeting signals are not easily predictable linear epitopes. Prior work located the CD81 targeting signals to the C-terminus and implicated palmitoylation and cholesterol binding for proper folding of CD81 in the membrane (55, 79, 80). These data are confirmed here, as CD81 was able to direct an S insert in the LEL from the native S pathway (into the lysosome) into the wild-type CD81 pathway first to the cell membrane and then on into EV. CD81 trafficking was dominant over S trafficking in this fusion molecule.

A fraction of any viral surface, including S protein, will serendipitously traffic into EV (81, 82). Tsai et al. used EV rather than artificial liposomes to deliver S mRNA (83). Others used EVs to carry ACE2 (84, 85). Different GFP variants have been prepended and appended to the two intracellular N- and C-terminal ends (56, 64, 86). Pegtel and colleagues succeeded in grafting a ph-sensitive reporter (pHluorin) into the SEL of CD63 (87); others were able to replace the LEL of CD63 with cyan fluorescent protein (88). The RBD domain when chemically conjugated to EV produced antibodies after inhalation (89). To our knowledge, no one has been able to insert an entire protein, particularly as large as a SARS-CoV-2 S, into any TSPAN and succeeded for the fusion protein to (i) trimerize and (ii) adopt the proper conformation, such evidenced here by RBD accessibility to ACE2 on purified recombinant CD81::S EVs.

This study deliberately focused on S protein trafficking. We used the induction of S-specific antibodies in mice as an assay to show that the homo-trimer correctly assembled on the EV (using a S trimer-specific ELISA), and the RBD was correctly folded and exposed to BCR binding (using an RBD-specific ELISA). To attain a clean result, no adjuvant was administered. Further studies are needed to delineate the magnitude and correlates of protection and to identify which adjuvant synergizes best with the physical nature of EVs. Unlike recombinant adenovirus vectors (Ad26.COV2.S) or pure protein complexes (NVX-CoV2373), EVs have a lipid component that may interact differently with alum or lipopolysaccharide-containing adjuvants. Such studies requiring *in vivo* BSL-3 containment are the next step.

## SUPPLEMENTAL MATERIAL

Supplemental File mbio.01922-24-s0001.docx Supplemental table and figures.

ASM does not own the copyrights to Supplemental Material that may be linked to, or accessed through, an article. The authors have granted ASM a non-exclusive, world-wide license to publish the Supplemental Material files. Please contact the corresponding author directly for reuse.
