# Supplementary material for: CD81 fusion alters SARS-CoV-2 Spike trafficking
Source: mBio. 2024 Aug 14;15(9):e01922-24. doi: 10.1128/mbio.01922-24 (PMC11389398; doi:10.1128/mbio.01922-24)
Supplement: Supplemental File — Supplemental table and figures. [file mbio.01922-24-s0001.docx]

**Supplemental Table 1**

**Constructs used in the manuscript.** Constructs are listed by the plasmid number, the backbone, and the name used in the paper. We also described mutations made to the Spike and CD81 proteins.

**
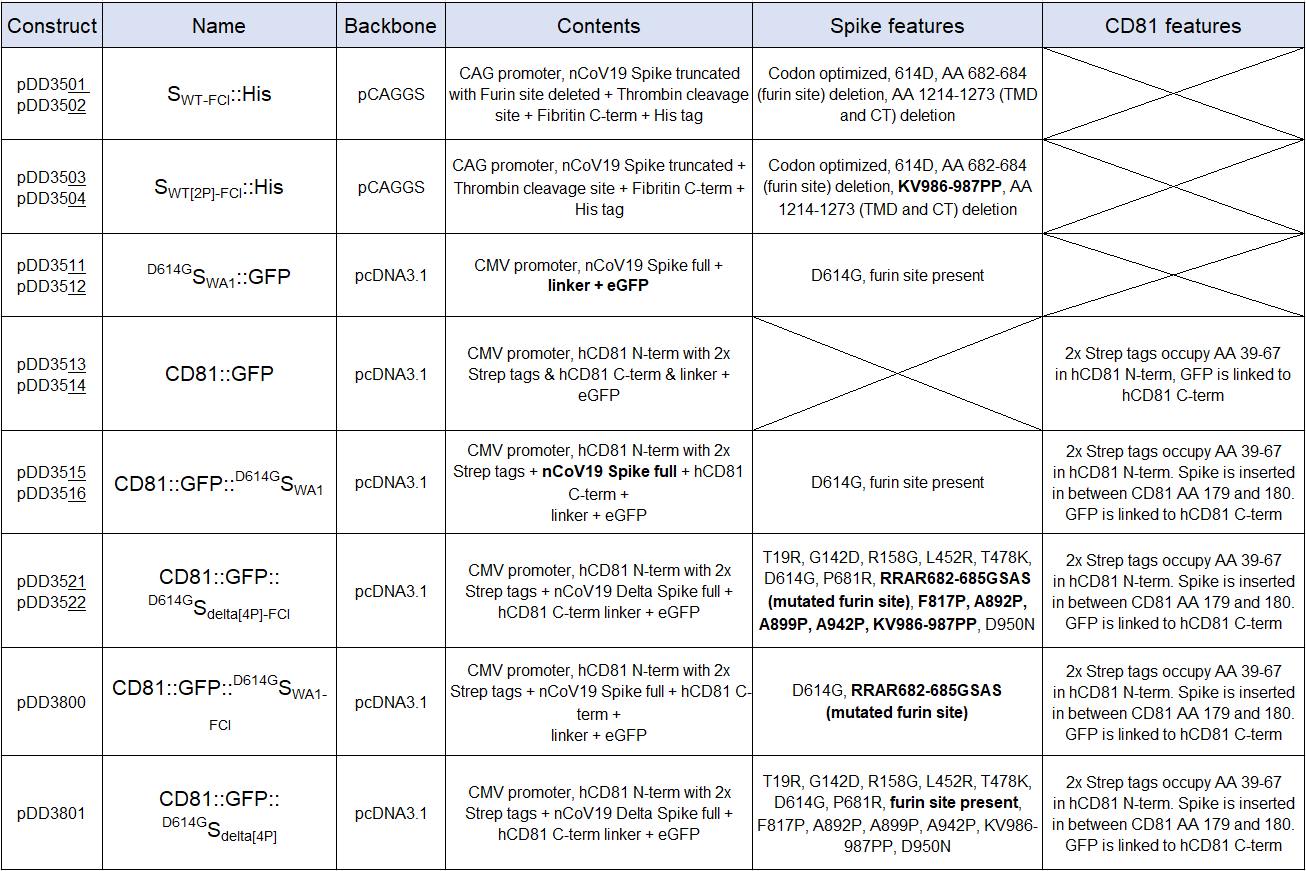
**


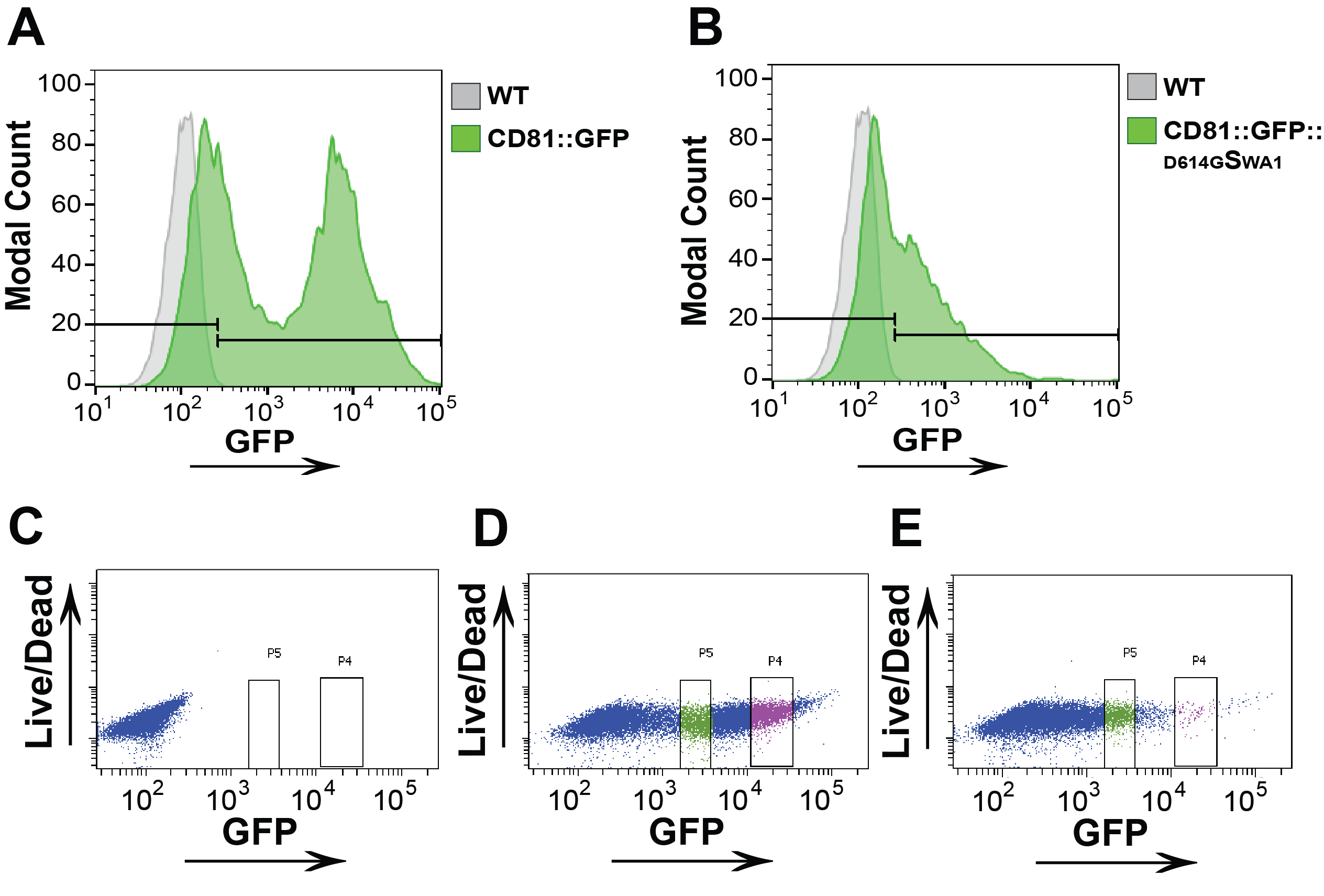


**Supplemental Figure 1:** Upon confluency, CD81-Strep-GFP, CD81-Strep-Spike-GFP, and U2OS WT cells were rinsed with Dulbecco's Phosphate Buffered Saline (Gibco, 14190-144) of equivalent media volume, followed by incubation with 0.05% Trypsin-EDTA (Gibco, Thermo Fisher, P: 25300-054) for 5 min at 37°C. One million cells were used for reading by flow cytometer. CD81-Strep-GFP **(A)** or CD81-Strep-Spike-GFP **(B)** cells were reported through GFP fluorescence using a FITC optical filter. U2OS WT cells were used as control. Clones with a single peak of FITC-H+ count were selected to establish permanent cell lines **(C-E)**.

**
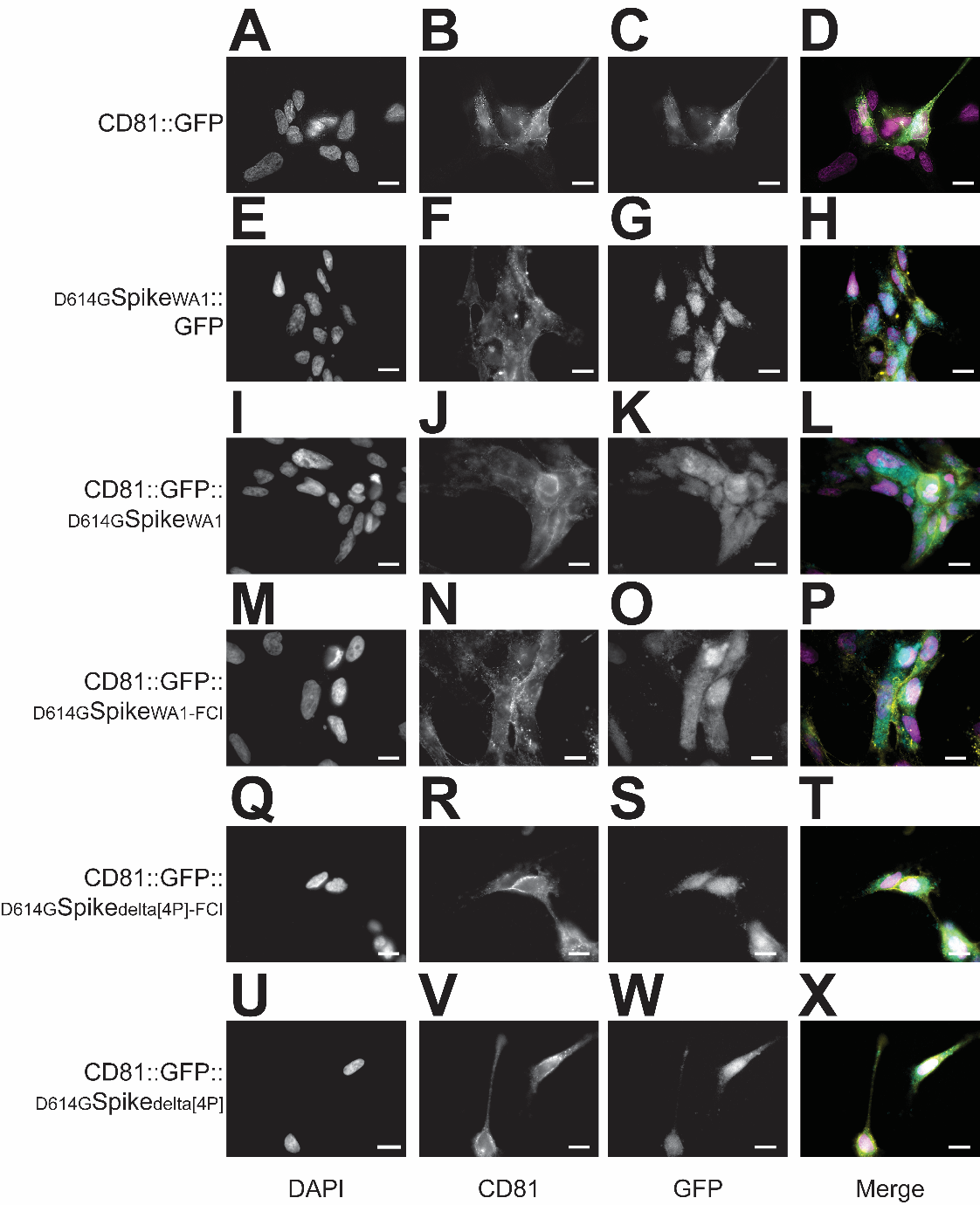
**

**Supplemental Figure 2:** The furin cleavage insert (FCI) does not alter the localization of the CD81-Spike-GFP cells. U2OS cells were transfected with CD81::GFP or various CD81::S::GFP constructs **(A-D)** No DNA control **(E-H)** CD81::GFP **(I-L)** CD81::GFP::^D614G^S_WA1_  **(M-P)** CD81::GFP::^D614G^S_WA1-FCI_ with the FCI removed (figure 4B) **(Q-T)** CD81-Stabilized Spike-GFP lacking the furin site (CD81::GFP::^D614G^S_delta[4P]-FC_) **(U-X)** CD81-Stablized Spike-GFP with the FCI re-inserted (CD81::GFP::^D614G^S_delta[4P]_ ), as described in Figure 4. Cells were fixed in methanol and then stained with CD81 primary antibody and an AlexaFluor 594 secondary antibody. Cells were then mounted and imaged using a Leica DM 5500B microscope with a 100X objective. Scale bar = 10 µm.

**
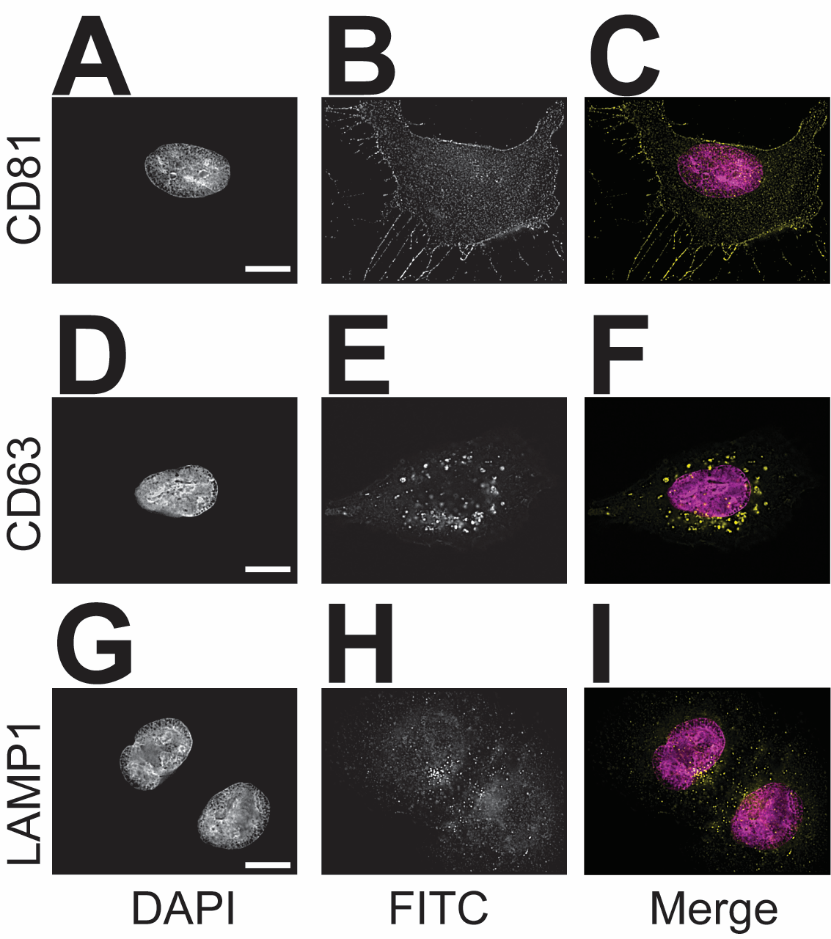
**

**Supplemental Figure 3:** Localization of subcellular markers in wild-type U2OS cells. Cells were fixed and stained with CD81 **(A-C)**, CD63 **(D-F)**, or LAMP1 **(G-I)**. After staining with the primary antibody, they were then stained with an AlexaFluor 594 secondary antibody and then imaged using a Leica DM 5500B microscope with a 100X objective. Scale bar = 10 µm.


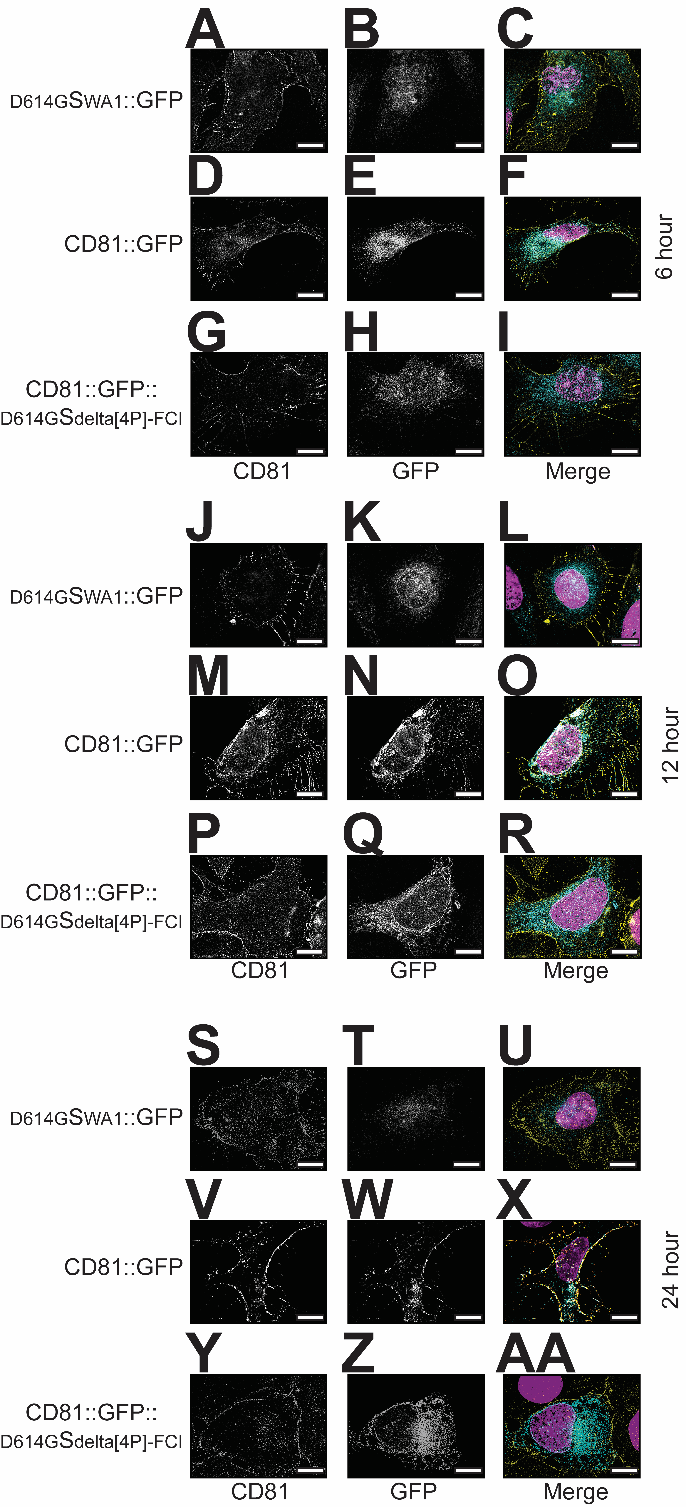


**Supplemental Figure 4:** Localization of CD81::GFP, S::GFP, or CD81::Spike::GFP mutants over time in comparison to endogenous CD81 expression. Cells were seeded, then transfected with different GFP constructs as previously described. Six, twelve, or twenty-four hours post-transfection, cells were fixed and stained with a CD81 primary antibody, then an AlexaFluor 594 secondary antibody and then imaged using a Leica DM 5500B microscope with a 100X objective. CD81 was used in this experiment as a plasma membrane marker. Scale bar = 10 µm.


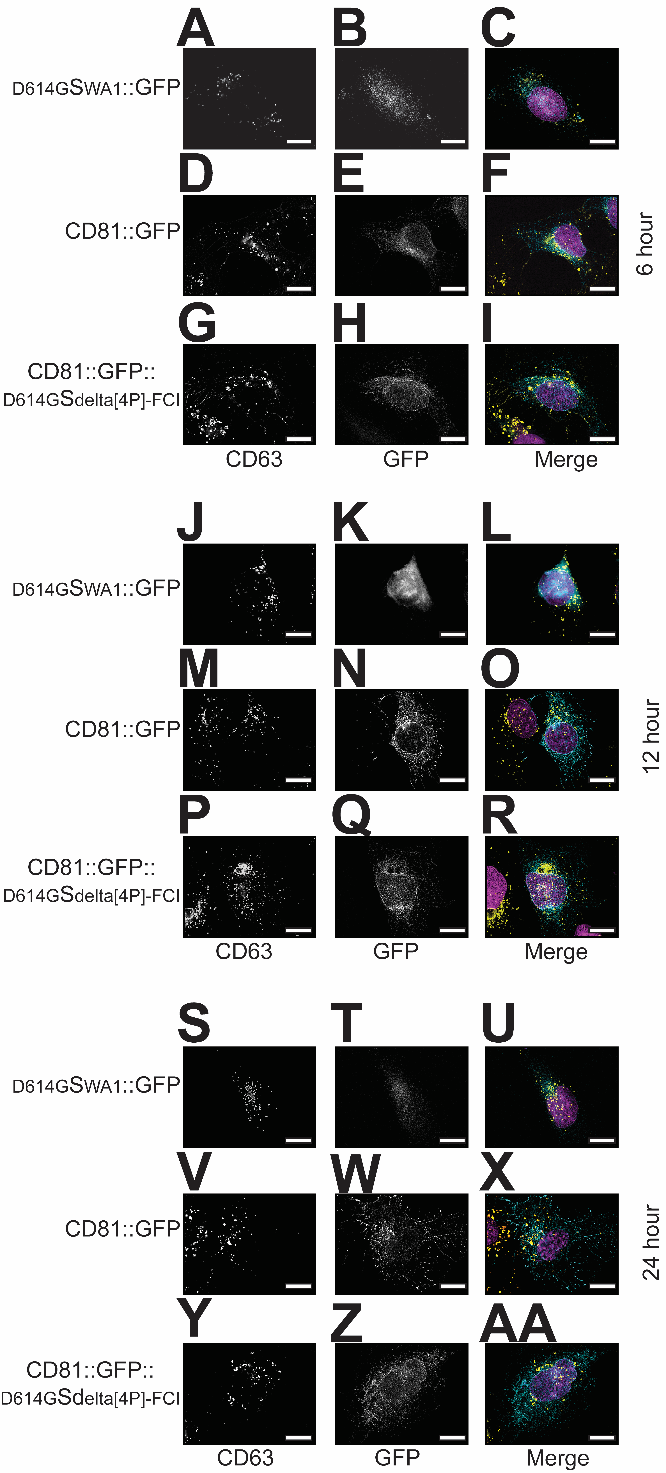


**Supplemental Figure 5:** Localization of CD81::GFP, S::GFP, or CD81::Spike::GFP mutants over time in comparison to endogenous CD63 expression. Cells were seeded, then transfected with different GFP constructs as previously described. Six, twelve-, or twenty-four-hours post-transfection, cells were fixed and stained with a CD63 primary antibody, then an AlexaFluor 594 secondary antibody and then imaged using a Leica DM 5500B microscope with a 100X objective. CD63 was used in this experiment as a multivesicular body marker. Scale bar = 10 µm.


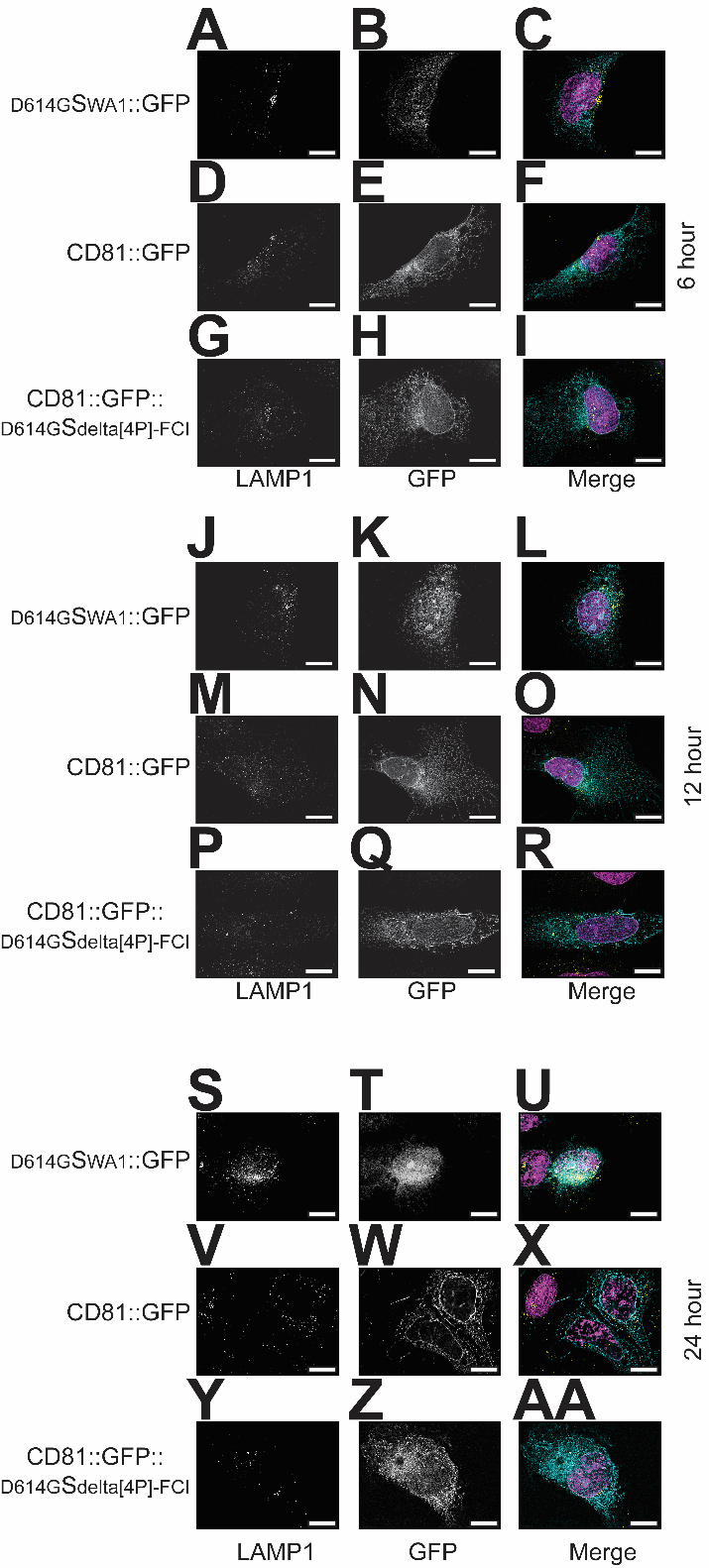


**Supplemental Figure 6:** Localization of CD81::GFP, S::GFP, or CD81::Spike::GFP mutants over time. Cells were seeded, then transfected with different GFP constructs as previously described. Six, twelve-, or twenty-four-hours post-transfection, cells were fixed and stained with a LAMP1 primary antibody, then an AlexaFluor 594 secondary antibody and then imaged using a Leica DM 5500B microscope with a 100X objective. LAMP1 was used in this experiment as a lysosome marker. Scale bar = 10 µm.


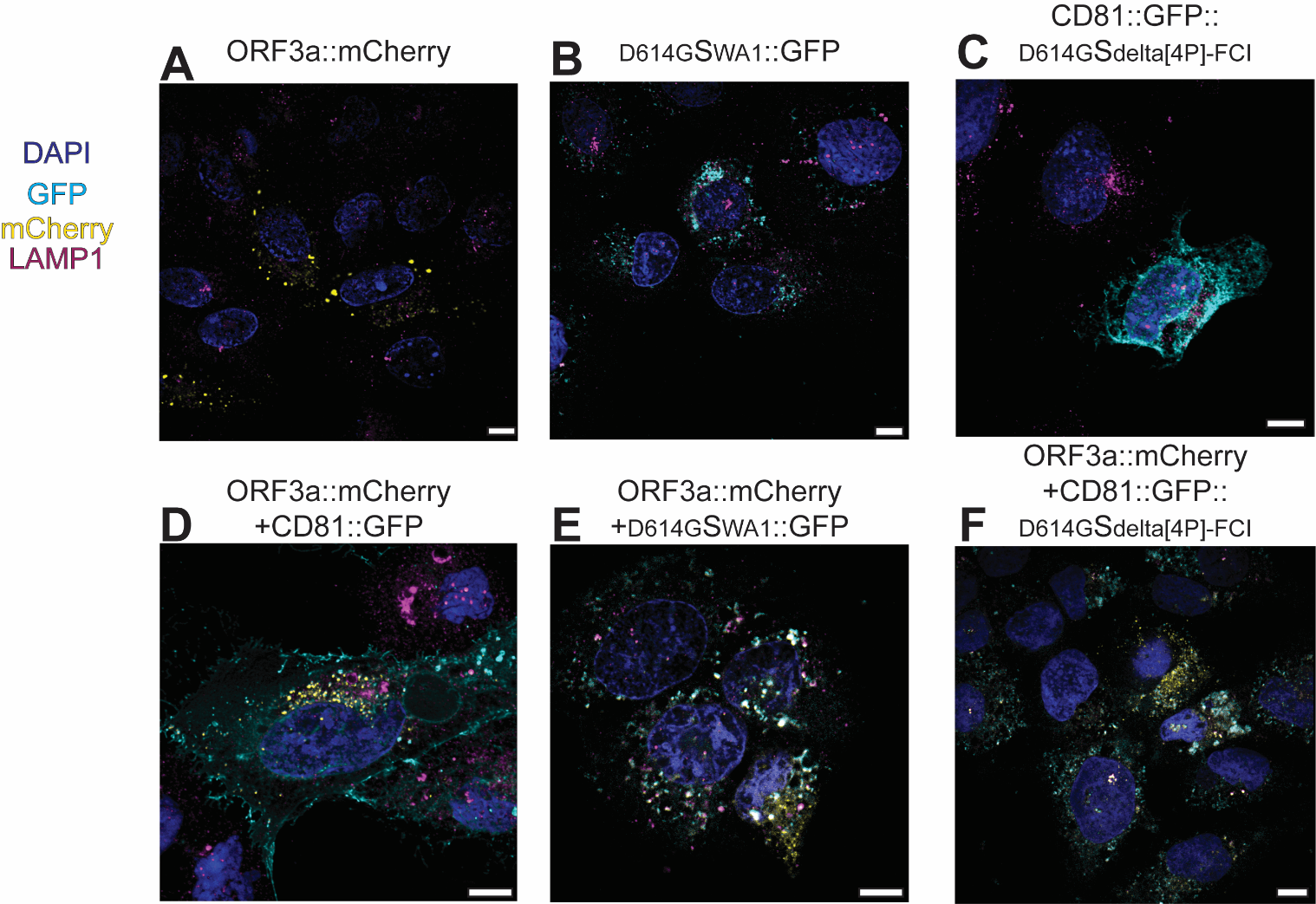


**Supplemental Figure 7:** Localization of CD81::GFP, S::GFP, or CD81::Spike::GFP constructs in the presence of SARS-CoV-2 orf3a. Cells were seeded onto glass coverslips. The next day the cells were transfected with orf3a::mCherry (**A**), S::GFP (**B**), CD81::S::GFP (**C**), or co-transfected with orf3a:mCherry and CD81::GFP (**D**), S::GFP (**E**), or CD81::S::GFP(**F**). Twenty-four hours post-transfection, cells were fixed with 100% MeOH at -20, then stained for the lysosome marker LAMP1 primary antibody, then an AlexaFluor 647 secondary antibody. Finally, cells were incubated with DAPI, then mounted on slides and imaged using an Olympus FV1000. Scale bar = 10 µm.


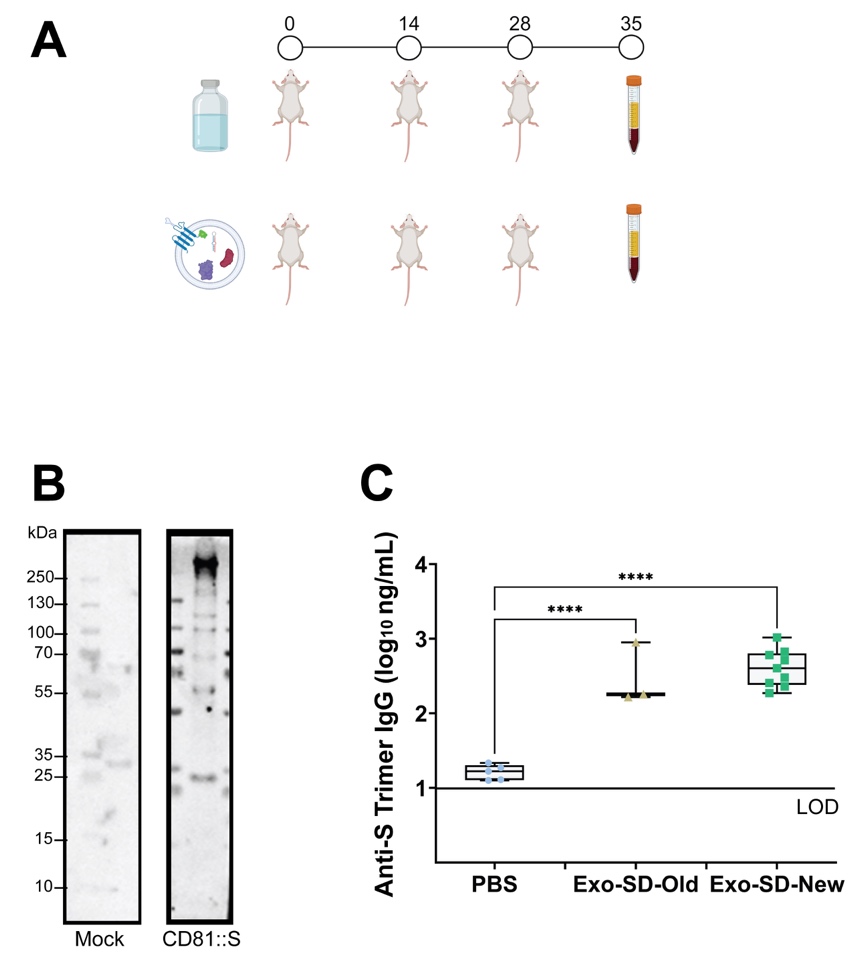


**Supplemental Figure 8:** Treatment with stabilized EV-S induces antibody formation. **(A)** Illustration of the treatment plan and boost regimen. The numbers on top indicate days of injection or (day 35) collection. The group size was n=5. **(B)** Western-Blot assay to test sera for the presence of anti-SARS-CoV-2 S antibodies. The target S_WT[2P]-FCI_::His was collected from conditioned cell media and purified using a His column. Protein was run on an SDS-PAGE gel. Serum from mice injected with PBS or EV-S was incubated with the membranes at 1:100 dilution and detected with anti-murine total IgG-conjugated to horseradish peroxidase. Weak bands on the left and numbers indicate molecular weight markers in kDa. **(C)** Results of an ELISA assay testing for the presence of trimer-Spike antibodies. This uses a commercial ELISA (Acro Biosystems RAS-T023). Shown is a box and whisker plot of range, median, 1st and 3rd quartile overlayed with individual data points for either S negative group (blue) or the S positive group (brown). The amount of anti-S specific IgG is shown on the vertical axis in ng/ml on a log 10 scale. Significance is indicated by the number of stars with ****: p≤0.0001 by one-way ANOVA with multiple comparisons.


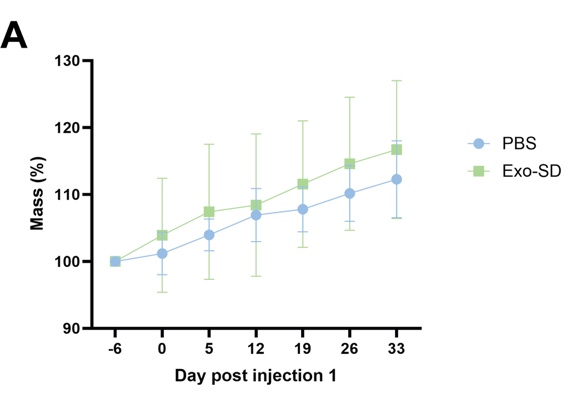


**Supplemental Figure 9:** Injections with EV-Spike did not cause deleterious effects on the mice. The animal facilities core took weight data for the mice during the different trials. Mice were weighed starting six days before injections started. The average weights were then graphed using GraphPad. There were no significant differences between the weights of different treatment groups. Shown are the mean and standard deviation.
